# Puupehenone, a Marine-Sponge-Derived Sesquiterpene Quinone, Potentiates the Antifungal Drug Caspofungin by Disrupting Hsp90 Activity and the Cell Wall Integrity Pathway

**DOI:** 10.1128/mSphere.00818-19

**Published:** 2020-01-08

**Authors:** Siddharth K. Tripathi, Qin Feng, Li Liu, David E. Levin, Kuldeep K. Roy, Robert J. Doerksen, Scott R. Baerson, Xiaomin Shi, Xuewen Pan, Wen-Hui Xu, Xing-Cong Li, Alice M. Clark, Ameeta K. Agarwal

**Affiliations:** a National Center for Natural Products Research, School of Pharmacy, University of Mississippi, Oxford, Mississippi, USA; b Department of Molecular and Cell Biology, Boston University Henry M. Goldman School of Dental Medicine, Boston, Massachusetts, USA; c Division of Medicinal Chemistry, Department of BioMolecular Sciences, School of Pharmacy, University of Mississippi, Oxford, Mississippi, USA; d Natural Products Utilization Research Unit, U.S. Department of Agriculture, Agricultural Research Service, Oxford, Mississippi, USA; e Verna and Marrs McLean Department of Biochemistry and Molecular Biology, Baylor College of Medicine, Houston, Texas, USA; f Division of Pharmacognosy, Department of BioMolecular Sciences, School of Pharmacy, University of Mississippi, Oxford, Mississippi, USA; g Division of Pharmacology, Department of BioMolecular Sciences, School of Pharmacy, University of Mississippi, Oxford, Mississippi, USA; Carnegie Mellon University

**Keywords:** Hsp90, caspofungin, cell wall integrity pathway, potentiation

## Abstract

The cell wall-targeting echinocandin antifungals, although potent and well tolerated, are inadequate in treating fungal infections due to their narrow spectrum of activity and their propensity to induce pathogen resistance. A promising strategy to overcome these drawbacks is to combine echinocandins with a molecule that improves their activity and also disrupts drug adaptation pathways. In this study, we show that puupehenone (PUUP), a marine-sponge-derived sesquiterpene quinone, potentiates the echinocandin drug caspofungin (CAS) in CAS-resistant fungal pathogens. We have conducted RNA sequencing (RNA-seq) analysis, followed by genetic and molecular studies, to elucidate PUUP’s CAS-potentiating mechanism. We found that the combination of CAS and PUUP blocked the induction of CAS-responding genes required for the adaptation to cell wall stress through the cell wall integrity (CWI) pathway. Further analysis showed that PUUP inhibited the activation of Slt2 (Mpk1), the terminal mitogen-activated protein (MAP) kinase in this pathway. We also found that PUUP induced heat shock response genes and inhibited the activity of heat shock protein 90 (Hsp90). Molecular docking studies predicted that PUUP occupies a binding site on Hsp90 required for the interaction between Hsp90 and its cochaperone Cdc37. Thus, we show that PUUP potentiates CAS activity by a previously undescribed mechanism which involves a disruption of Hsp90 activity and the CWI pathway. Given the requirement of the Hsp90-Cdc37 complex in Slt2 activation, we suggest that inhibitors of this complex would disrupt the CWI pathway and synergize with echinocandins. Therefore, the identification of PUUP’s CAS-potentiating mechanism has important implications in the development of new antifungal combination therapies.

**IMPORTANCE** Fungal infections cause more fatalities worldwide each year than malaria or tuberculosis. Currently available antifungal drugs have various limitations, including host toxicity, narrow spectrum of activity, and pathogen resistance. Combining these drugs with small molecules that can overcome these limitations is a useful strategy for extending their clinical use. We have investigated the molecular mechanism by which a marine-derived compound potentiates the activity of the antifungal echinocandin caspofungin. Our findings revealed a mechanism, different from previously reported caspofungin potentiators, in which potentiation is achieved by the disruption of Hsp90 activity and signaling through the cell wall integrity pathway, processes that play important roles in the adaptation to caspofungin in fungal pathogens. Given the importance of stress adaptation in the development of echinocandin resistance, this work will serve as a starting point in the development of new combination therapies that will likely be more effective and less prone to pathogen resistance.

## Abstract (precis)

Fungal infections cause more fatalities worldwide each year than malaria or tuberculosis. Currently available antifungal drugs have various limitations, including host toxicity, narrow spectrum of activity, and pathogen resistance. Combining these drugs with small molecules that can overcome these limitations is a useful strategy for extending their clinical use. We have investigated the molecular mechanism by which a marine-derived compound potentiates the activity of the antifungal echinocandin caspofungin. Our findings revealed a mechanism, different from previously reported caspofungin potentiators, in which potentiation is achieved by the disruption of Hsp90 activity and signaling through the cell wall integrity pathway, processes that play important roles in the adaptation to caspofungin in fungal pathogens. Given the importance of stress adaptation in the development of echinocandin resistance, this work will serve as a starting point in the development of new combination therapies that will likely be more effective and less prone to pathogen resistance.

## INTRODUCTION

Fungal infections affect an estimated 1.5 to 2 million people worldwide every year, exceeding those affected by either malaria or tuberculosis (1). The mortality rates associated with invasive fungal infections often exceed 50%, depending upon the pathogen and the geographical locations of infected populations (2). The major fungal pathogens responsible for causing these infections are species of Candida, Cryptococcus, and Aspergillus (2). Current antifungal drugs are inadequate in effectively treating these infections due to problems associated with host toxicity, resistance development, narrow spectrum of activity, and drug interactions (3). The echinocandins, the newest class of antifungals in clinical use, are lipopeptide molecules that inhibit the synthesis of β-1,3-d-glucan, a major component of the fungal cell wall (4). However, even though the echinocandins are more specific and less toxic than are the membrane-targeting antifungal agents such as azoles and polyenes, they still suffer from considerable drawbacks (5). The echinocandins have a narrow spectrum of activity and are strongly active mainly against *Candida* species. They are not fungicidal against *Aspergillus* spp. and are ineffective against *Cryptococcus* spp. In addition, the extensive use of echinocandins has resulted in the development of drug resistance in *Candida* species (4). One of the ways in which these limitations can be overcome is by combining an echinocandin drug with a bioactive compound that can either make the drug more effective or that can delay the onset of drug resistance (6). In addition, an understanding of the molecular targets inhibited by a drug-potentiating compound can provide new mechanistic insight into the cellular pathways involved in adaptation to the drug. For example, a new role for protein kinase C (PKC) signaling in azole resistance was identified when inhibitors of PKC signaling such as cercosporamide and staurosporine were found to potentiate azole activity in Candida albicans (7).

To identify echinocandin-potentiating molecules, we are currently investigating natural product compounds for their ability to improve the activity of the echinocandin drug caspofungin (CAS). Our investigation has identified a marine-sponge-derived sesquiterpene quinone named puupehenone (PUUP) that strongly potentiates CAS activity in fungal pathogens (see Fig. S1 in the supplemental material for the structure of PUUP). We have previously reported that PUUP exhibits antifungal activity against the major fungal pathogens C. albicans, Cryptococcus neoformans, and Aspergillus fumigatus (8). In addition to antifungal activity, PUUP also possesses antibacterial, antimalarial, antioxidant, and antitumor activities (9). The exact mechanism by which PUUP exerts these biological activities is not well understood. While one report has shown that the antioxidant activity of PUUP is due to lipoxygenase inhibition, another report indicated that it is due to the inhibition of membrane-bound NADPH oxidases (9). Studies on the antitumor activities of PUUP have suggested that it may target (i) glycogen-synthase kinase-3β, (ii) matrix metallopeptidase 2, and (iii) hypoxia-inducible factor-2 transcription (9). Thus, further mechanistic characterization of PUUP will facilitate the identification of its precise molecular targets as well as the mechanisms involved in its CAS potentiation effects.

10.1128/mSphere.00818-19.1 FIG S1 Schematic representation of compound structures and the cell wall integrity (CWI) pathway. (A) Structures of puupehenone, celastrol, and sapindoside A. (B) Signaling through the CWI pathway is initiated when cell wall (CW) damage, caused by CW-affecting agents such as CAS, is sensed by stress sensors Wsc1, Mid2, and Mtl1 in the plasma membrane (PM). These proteins bind to Rom2, a GDP/GTP exchange factor, which in turn activates Rho1, a GTP-binding protein. Rho1 activates the protein kinase Pkc1, which regulates a MAP kinase cascade. Pkc1 phosphorylates Bck1 (a MAP kinase kinase kinase), which in turn phosphorylates Mkk1 and Mkk2 (which are MAP kinase kinases). These two kinases finally activate the MAP kinase Slt2. This leads to phosphorylation of the transcription factor Rlm1, a key player in the CWI transcriptional program, which in turn activates cell wall biogenesis genes whose expression finally leads to cell wall repair. Download FIG S1, PDF file, 0.2 MB. Copyright © 2020 Tripathi et al. 2020 Tripathi et al. This content is distributed under the terms of the Creative Commons Attribution 4.0 International license.

In the present study, using Saccharomyces cerevisiae as a model organism, we have conducted RNA sequencing (RNA-seq) experiments followed by genetic and molecular analyses to gain insight into the mechanism behind the CAS-potentiating activity of PUUP. We found that exposure of yeast cells to a combination of PUUP and CAS prevented the induction of CAS-responding genes that are required for cell wall repair through the cell wall integrity (CWI) signaling pathway, a pathway required for adaptation to the cell wall damage exerted by cell wall-affecting agents such as CAS. Further studies revealed that PUUP inhibited the CWI pathway by inhibiting the activation of Slt2 (Mpk1), the terminal MAP kinase in this pathway. Our RNA-seq analysis also showed that PUUP, on its own, induced the expression of genes encoding chaperones and cochaperones involved in the heat shock response. Follow-up investigations revealed that PUUP interfered with the activity of the molecular chaperone Hsp90. Through molecular docking studies, we found that PUUP is predicted to occupy a binding site on Hsp90 that is involved in interactions between Hsp90 and its cochaperone protein Cdc37. Taken together, our results indicate that PUUP potentiates CAS activity by a mechanism that disrupts Hsp90 function as well as signaling through the CWI pathway.

## RESULTS

### PUUP potentiates CAS activity in fungal pathogens.

**FIG 1:**
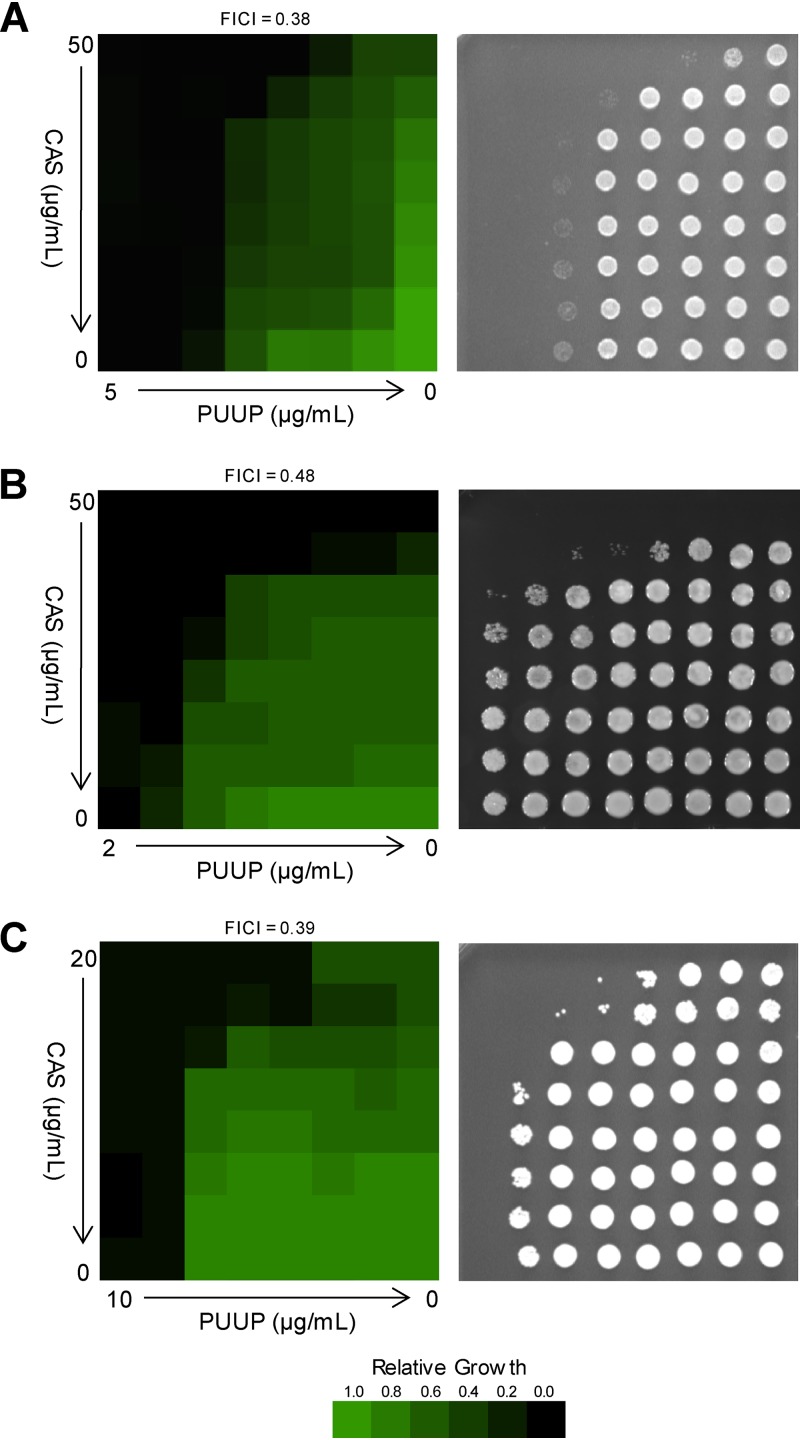
PUUP potentiates CAS activity in fungal pathogens. Dose matrix assays were performed to evaluate the combined effects of PUUP and CAS in fungal pathogens. Cells were grown in the presence of 2-fold serially diluted concentrations of PUUP and CAS for 48 h in microplates, and OD readings were measured. OD readings were normalized to the no-compound control well on each microplate. Data were quantitatively displayed using the TreeView software, and the resulting heat maps are shown in the left panels. To assess viability, 2-μl aliquots from each well were spotted on YPD agar plates, which were incubated for 24 h. Cell growth on the agar plates is shown in the right panels. The FICI value for each assay was calculated as described in Materials and Methods. Similar results were obtained in two independent experiments. (A) Dose matrix assay performed on Cryptococcus neoformans strain H99. Left, cell growth in broth; right, cell recovery on agar. (B) Dose matrix assay performed on Candida glabrata strain 102. Left, cell growth in broth; right, cell recovery on agar. (C) Dose matrix assay performed on Candida albicans strain DPL1010. Left, cell growth in broth; right, cell recovery on agar.

To evaluate the effect of PUUP on CAS activity, broth microdilution dose matrix assays were performed. In the CAS-insensitive pathogen C. neoformans, CAS alone exhibited poor activity, as expected; however, the combination of CAS and PUUP showed a synergistic effect (Fig. 1A, left), exhibiting a fractional inhibitory concentration index (FICI) of 0.38 (see Table S1 in the supplemental material for FICI calculations). When an aliquot of cells was spotted on yeast extract-peptone-dextrose (YPD) agar to assess their viability, cells from wells in which no growth was detected in the presence of both CAS and PUUP also exhibited no growth on the solid medium, indicating a fungicidal effect (Fig. 1A, right). In a CAS-resistant strain of Candida glabrata (strain no. 102 [10]), PUUP combined with CAS exhibited a synergistic effect, with an FICI of 0.48 (Fig. 1B, left). On the agar plate, a fungicidal effect was observed for cells exposed to 25 μg/ml CAS combined with either 1 μg/ml or 2 μg/ml PUUP (Fig. 1B, right). In a CAS-resistant strain of C. albicans (strain no. DPL1010 [11]), the combined effect of PUUP and CAS was strongly synergistic, exhibiting an FICI of 0.39 (Fig. 1C, left). On the agar plate, a fungicidal effect was observed for cells exposed to 20 μg/ml CAS combined with either 2.5 μg/ml or 5 μg/ml PUUP, as well as for cells exposed to 5 μg/ml PUUP combined with either 5 μg/ml CAS or 10 μg/ml CAS (Fig. 1C, right). Thus, PUUP demonstrated synergistic and fungicidal activity in combination with CAS in fungal pathogens with both inherent and acquired resistance to CAS.

10.1128/mSphere.00818-19.7 TABLE S1 Fractional inhibitory concentration index values for dose matrix assays. Download Table S1, PDF file, 0.02 MB. Copyright © 2020 Tripathi et al. 2020 Tripathi et al. This content is distributed under the terms of the Creative Commons Attribution 4.0 International license.

### Transcriptional responses to CAS, PUUP, and CAS+PUUP by RNA-seq analysis.

In this work, we conducted transcriptome as well as follow-up mechanistic studies in the model yeast S. cerevisiae due to the readily accessible databases of genome-wide studies, transcription factor interactions, and protein-protein interactions, as well as the availability of specific molecular and genetic tools relevant to this study. To ensure that PUUP also potentiates CAS activity in S. cerevisiae, we conducted a dose matrix assay in this organism (Fig. S2). Although the combined effect of CAS and PUUP (CAS+PUUP) was not synergistic in S. cerevisiae, we observed at least a 4-fold improvement in CAS activity in the presence of PUUP (Fig. S2). To identify the molecular pathways involved in PUUP’s CAS-potentiating effects, we conducted RNA-seq analysis with yeast cells that were exposed to CAS or PUUP at their respective 50% inhibitory concentrations (IC_50_s) for one doubling (∼4 h). Cells were also exposed to a combination of CAS and PUUP at the same concentrations for the same duration. Based on the dose matrix assays, we reasoned that a combined effect on cell growth would be expected to occur when each drug was present at its respective IC_50_. RNA-seq libraries prepared from the treated samples produced high-quality reads with a median quality score of Q > 30 and an average of 20 million reads per sample. Genes exhibiting significant differential expression (*P* < 0.01 and fold change of ≥2) between drug-treated and solvent-treated cells were identified (Table S2).

10.1128/mSphere.00818-19.2 FIG S2 PUUP potentiates CAS activity in Saccharomyces cerevisiae. A dose matrix assay was performed to evaluate the combined effects of PUUP and CAS in S. cerevisiae. Cells were grown in SD broth (MOPS buffered [pH 7.0]) in the presence of 2-fold serially diluted concentrations of PUUP or CAS, and optical density (OD) readings were measured on a microplate reader after 48 h. OD readings were normalized to the no-compound control. Data were quantitatively displayed using the TreeView software, and the resulting heat map is shown. Download FIG S2, PDF file, 0.01 MB. Copyright © 2020 Tripathi et al. 2020 Tripathi et al. This content is distributed under the terms of the Creative Commons Attribution 4.0 International license.

10.1128/mSphere.00818-19.8 TABLE S2 RNA-seq-based transcript profiles of yeast cells exposed to CAS, PUUP, and CAS+PUUP. Download Table S2, XLSX file, 0.1 MB. Copyright © 2020 Tripathi et al. 2020 Tripathi et al. This content is distributed under the terms of the Creative Commons Attribution 4.0 International license.

**FIG 2:**
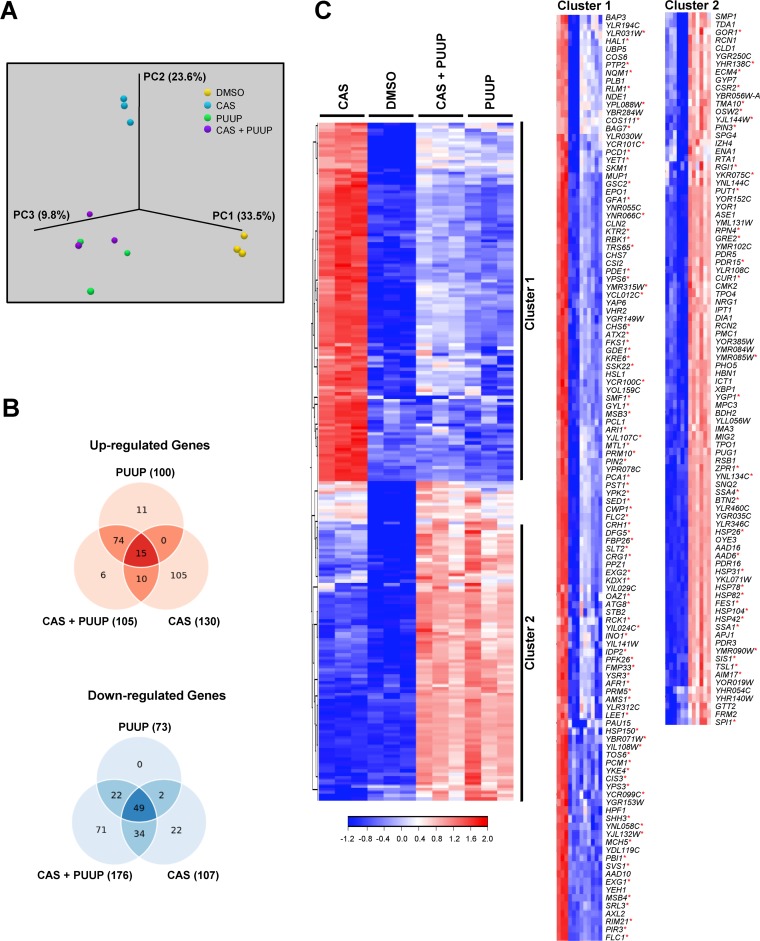
RNA-seq analysis of yeast cells treated with CAS, PUUP, or CAS+PUUP. (A) The experimental variation between RNA-seq experiments performed on 3 biological replicate samples was analyzed with a PCA plot. (B) Venn diagrams comparing the number of up- or downregulated genes that significantly responded (≥2-fold induction, *P* < 0.01) to the 3 treatments. (C) Hierarchical cluster analysis was performed on 221 genes that displayed a ≥2-fold induction (*P* < 0.01) in response to at least one of the three treatments compared to the DMSO control. Clustering was performed on normalized FPKM values using Euclidean distance and complete linkage settings. Clusters 1 and 2 are expanded on the right to display the names of genes within each cluster. Red asterisks indicate genes identified in the YEASTRACT database that are known to be induced by the transcription factors Rlm1 (CWI pathway regulator) and Hsf1 (heat shock response regulator) in clusters 1 and 2, respectively.

Principal-component analysis (PCA) revealed that the RNA-seq samples within each treatment group clustered together and that there was high reproducibility among the three replicate samples in each group (Fig. 2A). Interestingly, the samples treated with PUUP and with CAS+PUUP clustered together, indicating that the transcriptional responses to PUUP alone and to the combination of CAS+PUUP were similar to each other (Fig. 2A). This result was also evident in the distribution of the number of genes that responded to each treatment (Fig. 2B). Among the upregulated genes, the largest overlap of 74 genes was seen in the transcriptional response to PUUP and to CAS+PUUP (Fig. 2B). A set of 105 genes were upregulated in response to CAS only, further indicating that the response to CAS is different from the response to PUUP and to CAS+PUUP (Fig. 2B). Among the downregulated genes, a substantial number of genes (49 genes) commonly responded to all three treatments, indicating that the majority of the downregulated genes responded similarly across the three treatments (Fig. 2B).

To further visualize the transcriptional relatedness among the samples, we performed hierarchical cluster analysis on a set of 221 genes that were induced in response to at least one of the three treatments (Fig. 2C). Two clusters of genes were clearly evident. Cluster 1 consisted of 117 genes that were strongly induced by CAS and not by either PUUP or CAS+PUUP (Fig. 2C). Cluster 2 consisted of 90 genes that were strongly induced by PUUP and by CAS+PUUP but not by CAS (Fig. 2C). As expected, cluster 1 primarily consisted of genes that are known to respond to the cell wall damage exerted by drugs such as CAS and that play a role in cell wall biogenesis and the CWI signaling pathway (12, 13). Genes encoding cell wall proteins (e.g., *CWP1*, *CRH1*, and *PIR3*) as well as genes required for cell wall synthesis (e.g., *FKS1* and *GSC2*) and CWI signaling (e.g., *SLT2* and *MLP1* [*KDX1*]) were found in cluster 1, and these genes are known to be mainly regulated by the transcription factor Rlm1 (14, 15). Based on the YEASTRACT database, a repository of associations between yeast transcription factors and their target genes (16), 83 Rlm1-regulated genes were found to be present in cluster 1 (marked by asterisks in Fig. 2C). The upregulation of these genes was not evident when cells were exposed to CAS plus PUUP, indicating that PUUP disrupted the CAS-mediated induction of the cell wall stress response pathway (Fig. 2C). In addition, PUUP alone did not induce these genes, suggesting that PUUP’s mechanism of action (MOA) does not involve cell wall damage. Instead, PUUP caused the induction of numerous genes, including chaperones and cochaperones, involved in the heat shock response (Fig. 2C, cluster 2), which is mainly regulated by the transcription factor Hsf1 (17). Based on the YEASTRACT database (16), 35 genes known to be induced by Hsf1 were found to be present in cluster 2 (marked by asterisks in Fig. 2C). Interestingly, these genes continued to be upregulated when cells were exposed to PUUP+CAS, indicating that CAS did not interfere with the transcriptional response to PUUP (Fig. 2C).

### PUUP disrupts the CWI signaling pathway.

The fungal CWI pathway is the major signaling pathway that regulates cell wall repair in response to cell wall stress (18). This pathway is initiated when cell wall stress is monitored by cell surface proteins that contain highly *O*-mannosylated extracellular domains, which act as rigid sensors of the extracellular matrix. These proteins mediate nucleotide exchange on the small G protein Rho1, which leads to the activation of protein kinase C encoded by *PKC1*. Pkc1 activates a MAP kinase cascade, which relays the cell wall stress signal to the nucleus (Fig. S1). The terminal MAP kinase in this cascade is Slt2, whose activation leads to the phosphorylation of Rlm1, one of the major transcription factors required for the activation of cell wall biogenesis genes. Signaling through this pathway can be monitored either by measuring the activity of a *lacZ* reporter driven by Rlm1-responsive promoters or by detecting the phosphorylation of Slt2 using an anti-phospho-p42/p44 antibody (18). We made use of both of these assays to confirm that PUUP disrupts signaling through the CWI pathway.

**FIG 3:**
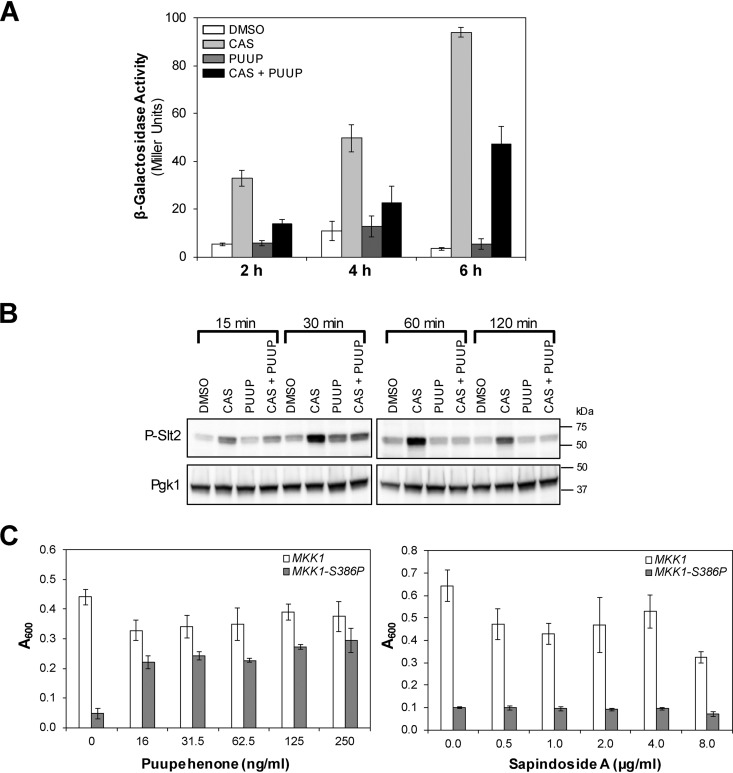
PUUP interferes with the CWI pathway. (A) β-Gal activity was measured in yeast cells harboring a construct containing the promoter region of *MLP1* fused to *lacZ*. Cells were treated with DMSO, CAS, PUUP, or CAS+PUUP for 2 h, 4 h, and 6 h. DMSO treatment was at 0.5% and compound treatments were at their respective IC_50_s. Data are represented as the mean ± SD for triplicate samples. (B) Western analysis of phosphorylated Slt2 was conducted with WT yeast cells (strain S288C) that were treated with DMSO, CAS, PUUP, or CAS+PUUP for the indicated time periods. DMSO treatment was at 0.5%, and compound treatments were at their respective IC_50_s. The constitutively expressed Pgk1 was measured as a loading control. Similar results were obtained in two independent experiments. (C) Growth in the presence of various subinhibitory concentrations of PUUP was compared between a strain expressing WT *MKK1* (open bars) and a mutant strain expressing a constitutive allele of *MKK1* (*MKK1-S386P*; closed bars). As a control, growth was measured in the presence of sapindoside A, a compound that also potentiates CAS activity. Data are represented as the mean ± SD for triplicate samples.

First, we used a construct in which *lacZ* is driven by the promoter of *MLP1* (*KDX1*), a gene that contains an Rlm1-binding site in its promoter that strongly responds to cell wall stress (19). Yeast cells containing this construct were exposed to dimethyl sulfoxide (DMSO), CAS, PUUP, or CAS+PUUP, and β-galactosidase (β-Gal) activity was measured. As expected, CAS treatment resulted in the induction of β-Gal activity compared to DMSO treatment, with maximum induction occurring at 6 h after treatment (Fig. 3A). PUUP treatment did not show any significant induction in β-Gal activity, in agreement with its transcript profile indicating that PUUP does not induce cell wall damage (Fig. 3A). Treatment of cells with CAS in the presence of PUUP diminished the β-Gal induction that was observed in cells treated with CAS alone, confirming that the presence of PUUP prevents the CAS-mediated induction of the CWI pathway (Fig. 3A).

In addition, we analyzed Slt2 phosphorylation in protein extracts prepared from wild-type yeast cells treated with DMSO, CAS, PUUP, or CAS+PUUP. As expected, CAS treatment resulted in Slt2 phosphorylation (13) within 15 min of exposure (Fig. 3B). Phosphorylation of Slt2 was observed up to 60 min and was slightly reduced at the 120-min time point. PUUP treatment did not show any significant increase in Slt2 phosphorylation (Fig. 3B). Interestingly, CAS treatment in the presence of PUUP resulted in diminished Slt2 phosphorylation at 15 min and 30 min and almost no phosphorylation at 60 min and 120 min (Fig. 3B). These results further confirm that PUUP inhibits the CAS-mediated activation of the CWI pathway.

Finally, we made use of a yeast mutant that activates the CWI pathway constitutively. This mutant strain carries a constitutively active allele of *MKK1* (*MKK1-S386P*), which causes growth arrest when overexpressed under the inducible control of the *GAL1* promoter (20). In this mutant, the CWI pathway is hyperactivated through the overactivation of Slt2 and Rlm1, which act downstream from Mkk1 in the pathway (Fig. S1). This growth-inhibitory effect is suppressed by deletion of the *SLT2* gene or by a chromosomal mutation in the *RLM1* gene (20). Therefore, a compound that suppresses the growth defect of this mutant would likely inhibit the CWI pathway downstream of Mkk1. We grew this strain as well as a strain expressing the wild-type *MKK1* gene in the presence of various subinhibitory concentrations of PUUP. Doses of PUUP up to 250 ng/ml did not affect the growth of the wild-type *MKK1*-expressing strain (Fig. 3C). The *MKK1-S386P*-expressing strain exhibited growth arrest in the absence of PUUP (Fig. 3C). However, in the presence of PUUP, there was a dramatic increase in growth, indicating that PUUP suppressed the growth defect of this mutant (Fig. 3C). We also tested in this assay another natural product compound, sapindoside A, which was also identified in our studies as a potentiator of CAS activity in fungal cells (Fig. S1 and S3). This compound did not suppress the growth defect of the mutant allele-expressing strain, indicating that the growth suppression effect is specific to PUUP (Fig. 3C). These results demonstrate that PUUP disrupts the CWI pathway downstream of Mkk1.

10.1128/mSphere.00818-19.3 FIG S3 Sapindoside A potentiates CAS activity in fungal cells. Dose matrix assays were performed to evaluate the combined effects of CAS and sapindoside A (SAPI). Cells were grown in the presence of 2-fold serially diluted concentrations of CAS or SAPI for 48 h in microplates, and OD readings were measured on a microplate reader. OD readings were normalized to the no-compound control well on each microplate. Data were quantitatively displayed using the TreeView software, and the resulting heat maps are shown in the left panels. To assess viability, 2-μl aliquots from each well were spotted on YPD agar plates, which were incubated for 24 h. Cell growth on the agar plates is shown in the right panels. (A) Dose matrix assay performed on Candida glabrata strain 102, a CAS-resistant clinical isolate. (B) Dose matrix assay performed on Candida albicans strain DPL1009, a CAS-resistant clinical isolate. (C) Dose matrix assay performed on Saccharomyces cerevisiae. Download FIG S3, PDF file, 0.05 MB. Copyright © 2020 Tripathi et al. 2020 Tripathi et al. This content is distributed under the terms of the Creative Commons Attribution 4.0 International license.

### PUUP interferes with Hsp90 activity.

**FIG 4:**
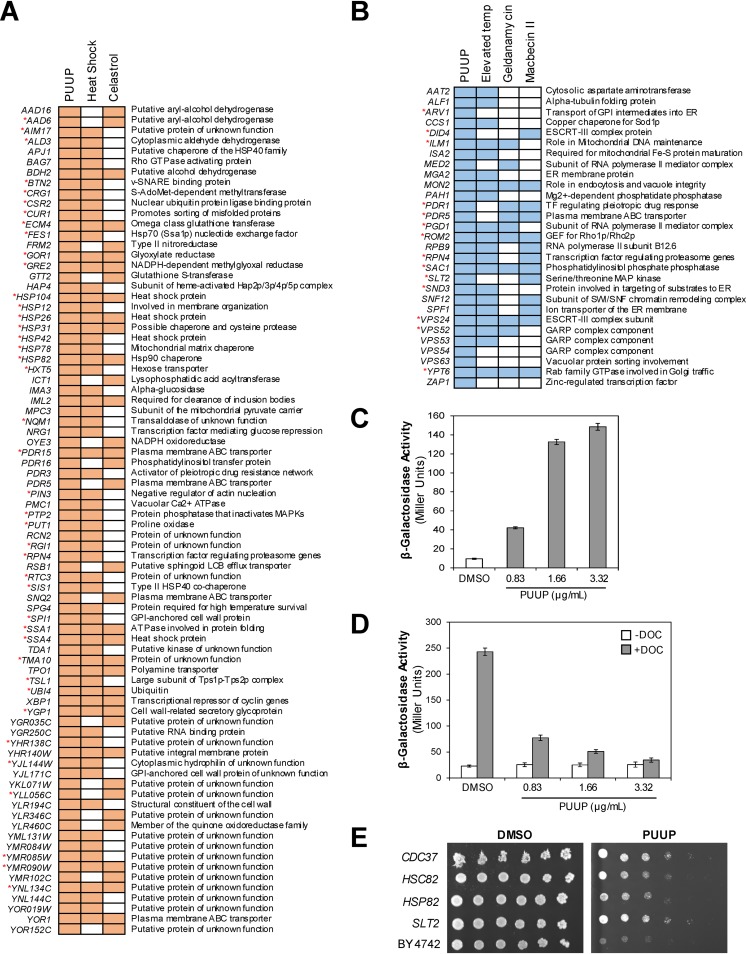
PUUP interferes with Hsp90 activity. (A) Comparison of the transcript profile of PUUP with previously reported transcript profiles for the response to heat shock and the Hsp90 inhibitor celastrol. A diagrammatic representation of the comparison is shown for 80 genes that were induced by PUUP and also induced by either heat shock or celastrol. Colored boxes indicate upregulated genes. Red asterisks indicate genes identified in the YEASTRACT database that are induced by Hsf1. (B) Comparison of the 29 mutants identified in the genome-wide fitness profile of PUUP with previously reported mutants displaying hypersensitivity to either elevated temperature or the Hsp90 inhibitors geldanamycin and macbecin II. A diagrammatic representation of the comparison is shown, with colored boxes indicating hypersensitive mutants. Genes deleted in the mutants are listed on the left. Red asterisks indicate genes which encode proteins that interact with the yeast Hsp90 isoforms Hsp82 and Hsc82, as reported in the BioGRID database. (C) WT yeast cells containing an HSE-*lacZ* reporter were treated with DMSO or PUUP for 4 h, and β-Gal activity was measured. Values shown are the mean ± SD from triplicate samples. (D) WT yeast cells transformed with a glucocorticoid receptor assay system were treated with DMSO or PUUP along with 20 μM DOC or vehicle for 2 h, and β-Gal activity was measured. Values shown are the mean ± SD from triplicate samples. (E) Fivefold dilutions of parent strain BY4742 and strains containing plasmids overexpressing *CDC37*, *HSC82*, *HSP82*, and *SLT2* were inoculated on agar plates containing DMSO or PUUP and incubated for 3 days. Two independent transformant colonies gave similar results. v-SNARE, vesicle SNAP REceptor; MAPK, mitogen-activated protein kinase; LCB, long-chain base; GPI, glycophosphatidylinositol; ER, endoplasmic reticulum; ESCRT, endosomal sorting complexes required for transport; TF, transcription factor; GEF, guanine nucleotide exchange factor; SWI/SNF, SWItch/Sucrose NonFermentable; GARP, Golgi-associated retrograde protein.

To gain insight into the MOA of PUUP, we further analyzed its transcriptional response by GO term enrichment analysis (Table 1). The overrepresented functional categories (*P* ≤ 0.05) for PUUP-induced genes included “protein folding” and “response to heat,” and these categories included several genes encoding chaperones, such as heat shock proteins (*HSP104*, *HSP12*, *HSP26*, *HSP78*, and *HSP82*), regulators of heat shock proteins (*APJ1*, *SSA1*, and *SSA4*), and cochaperones (*SIS1*). We also compared PUUP’s transcriptional response (Fig. 4A) to that generated by heat shock previously reported by Gasch et al. (21), in which 10 different heat shock treatments were examined. We found that 63 PUUP-induced genes also responded to 3 or more heat shock treatments in the Gasch et al. study, and the majority of these genes are induced by Hsf1, based on the YEASTRACT database (marked by asterisks in Fig. 4A). Thus, PUUP’s transcriptional response is similar to the heat shock response.

**TABLE 1:** Functional distribution of PUUP-responding genes^*a*^

GO term	PUUP data set frequency (%)	Genome frequency (%)	*P* value	Genes annotated to the GO term
Upregulated genes				
Response to chemicals	28	8.88	2.22E−08	*ECM4, FES1, FRM2, HAP4, HBN1, HSP104, HSP12, HSP31, IML2, MIG2, NQM1, NRG1, PDR15, PDR16, PDR3, PDR5, RPN4, SIS1, SNQ2, SSA1, SSA4, TDA1, TPO1, XBP1, YJL144W, YKL071W, YNL134C, YOR1*
Transmembrane transport	12	7.32	3.09E−02	*BAP2, HXT5, MPC3, PDR15, PDR5, PMC1, PUG1, SNQ2, SSA1, SSA4, TPO1, YOR1*
Protein folding	10	1.75	9.52E−06	*APJ1, BTN2, CUR1, HSP104, HSP26, HSP78, HSP82, SIS1, SSA1, SSA4*
Response to heat	8	1.4	7.51E−05	*CUR1, HSP104, HSP12, HSP26, HSP78, HSP82, SSA1, SSA4*
Response to oxidative stress	8	1.91	5.59E−04	*FRM2, HBN1, HSP104, HSP12, HSP31, NQM1, TDA1, XBP1*
Downregulated genes				
Conjugation	25.71	1.91	9.74E−16	*AFB1, AGA1, FAR1, FIG1, FIG2, FUS1, FUS2, FUS3, GPA1, KAR4, MF(ALPHA)1, MF(ALPHA)2, PRM1, PRM3, SAG1, SST2, STE2, STE3*
Response to chemical	20	8.88	2.01E−03	*AFB1, AGA1, FAR1, FIG2, FUS3, GPA1, KAR4, MF(ALPHA)1, MF(ALPHA)2, SAG1, SAM3, SST2, STE2, STE3*
Transposition	12.86	1.77	3.73E−06	*FUS3, TEC1, YAR010C, YBL005W-A, YDR365W-B, YJR027W, YOL103W-B, YPR137C-A, YPR158C-D*
Transmembrane transport	12.86	7.32	3.80E−02	*FTR1, HXT4, HXT7, POR1, PRM6, SAM3, YCT1, ZRT1, ZRT2*
Signaling	11.43	5.62	2.60E−02	*FUS2, FUS3, GPA1, MF(ALPHA)1, MF(ALPHA)2, SST2, STE2, STE3*
DNA recombination	8.57	3.93	3.71E−02	*NDJ1, SHU1, YDR365W-B, YJR027W, YOL103W-B, YPR158C-D*

a Data (100 upregulated genes and 73 downregulated genes) were organized into Gene Ontology (GO)-based biological process categories using the GO Term Mapper tool (https://go.princeton.edu/cgi-bin/GOTermMapper). Significantly overrepresented categories (*P* ≤ 0.05) with a PUUP data set frequency of ≥8% are listed.

An interesting feature of Hsf1 regulation is that heat shock proteins such as Hsp90 act as repressors of Hsf1, and pharmacological inhibition of Hsp90 results in the activation of Hsf1 (17). Therefore, we compared the transcriptional response of PUUP with that of the Hsp90 inhibitor celastrol (22), whose transcriptional profile generated in S. cerevisiae was available for comparison (23). We found that 38 genes were commonly induced by both PUUP and celastrol (Fig. 4A). Interestingly, celastrol is a plant-derived triterpene (24) containing a quinone methide structural moiety which is also present in PUUP (25) (see Fig. S1 for the structures of PUUP and celastrol). The similarities in the transcriptional profiles of PUUP and celastrol suggest that the two compounds might have similar MOAs.

To further identify the molecular pathways targeted by PUUP, we conducted a genome-wide fitness profile analysis (26) in which we screened a whole-genome collection of pooled haploid yeast deletion mutants against PUUP. Upon individual validation, we identified 29 mutants that were significantly more sensitive to PUUP than was the wild-type strain (Fig. 4B). Given our observations on PUUP’s transcript profile, we compared PUUP’s fitness profile with the fitness profiles that have been reported for the response to elevated temperature and to the Hsp90 inhibitors geldanamycin and macbecin II in S. cerevisiae (27–29). Of the 29 PUUP-hypersensitive mutants, 20 mutants were also hypersensitive to elevated temperature, 11 mutants were also hypersensitive to geldanamycin, and 13 mutants were also hypersensitive to macbecin II (Fig. 4B). The statistical significance of these comparisons was determined by Fisher’s exact test, and in each of the three comparisons, the overlap with the PUUP data set was found to be statistically significant (*P < *0.02). Thus, there are considerable similarities between the mutants associated with sensitivity to PUUP, geldanamycin, macbecin II, and increased heat. It is worth noting that the majority of the PUUP-hypersensitive mutants carried deletions in genes required for protein trafficking (Fig. 4B), consistent with the reported role of Hsp90 in various aspects of the secretory pathway (27). We also found that 14 of the 29 mutants identified in our study contained deletions in genes encoding proteins that are known to interact with the yeast Hsp90 isoforms Hsp82 and Hsc82 (marked by asterisks in Fig. 4B), based on the BioGRID database (30). Interestingly, many of these have been identified to serve as Hsp90 client proteins in S. cerevisiae, including Did4, Pdr1, SacI, Slt2, Vps24, and Ypt6 (31).

Next, we conducted studies to confirm that PUUP induces a heat shock response by mediating Hsf1 activation. Yeast Hsf1, upon activation, binds to heat shock elements (HSEs) in the promoter regions of heat shock response target genes (17). To monitor this activation, we made use of a construct in which the promoter of *SSA3*, a heat shock target gene, is fused to *lacZ* (32). Yeast cells containing this construct were exposed to either DMSO or PUUP, and β-Gal activity was measured. We observed an increase in β-Gal activity with increasing PUUP concentration, compared to the DMSO control, indicating Hsf1 activation (Fig. 4C). We also observed this activation in response to celastrol (Fig. S4A), consistent with the observation that Hsp90 inhibitors activate Hsf1 (17). A reporter construct carrying a mutation in the HSE promoter was not induced by PUUP or celastrol treatment, confirming that reporter activation required a functional HSE (Fig. S4A). These results further demonstrate the mechanistic similarities between PUUP and celastrol.

10.1128/mSphere.00818-19.4 FIG S4 Mutated versions of HSE-*lacZ* and the glucocorticoid receptor assay system do not respond to PUUP and celastrol (CELA). (A) Yeast cells containing different versions of the HSE-*lacZ* reporter were treated with DMSO (0.25%), PUUP (0.9 μg/ml), or CELA (9 μg/ml) for 4 h, and β-galactosidase (β-Gal) activity was measured. To maintain the solubility of CELA, 50 mM Tris-HCl [pH 7.5] was added to the CELA-treated cultures. CELA was purchased from Cayman Chemical Company (Ann Arbor, MI). Values shown are the mean ± standard deviation (SD) from triplicate samples. Cells containing the construct with the wild-type version of the HSE promoter respond to PUUP and CELA (left), while cells containing the construct with a mutation at position −156 of the HSE promoter, which disrupts its activation by Hsf1 (Boorstein and Craig [32]), do not respond to PUUP and CELA (right). (B) Yeast cells transformed with different versions of the glucocorticoid receptor (GR) assay system were treated with DMSO (0.25%), PUUP (1.66 μg/ml), or CELA (4.5 μg/ml) along with 20 μM DOC or vehicle for 2 h, and β-Gal activity was measured. Values shown are the mean ± SD from triplicate samples. Left, data generated with yeast cells transformed with the wild-type version of the GR assay system (consisting of plasmids p413GPD-rGR and pYRP-GRE*lacZ*). The addition of DOC activated reporter activity, and the presence of PUUP or CELA inhibited this activation. Right, data generated with yeast cells transformed with a mutated version of the GR assay system (consisting of plasmids pHCA-N525 and pYRP-GRE*lacZ*). The pHCA-N525 plasmid carries a truncated form of GR, up to amino acid 525, that excludes the ligand binding domain and functions as an Hsp90-independent constitutive activator (Godowski et al. [34]). High levels of β-Gal activity were observed in these cells under –DOC as well as +DOC conditions, and this activity was not affected by the presence of PUUP or CELA, indicating that the two compounds specifically interfere with Hsp90 activity. Download FIG S4, PDF file, 0.3 MB. Copyright © 2020 Tripathi et al. 2020 Tripathi et al. This content is distributed under the terms of the Creative Commons Attribution 4.0 International license.

To more directly evaluate whether PUUP modulates Hsp90 function, we made use of an Hsp90 functional assay system in yeast cells that monitors the activation of the mammalian glucocorticoid receptor (GR), a well-characterized Hsp90 client protein (33). This assay system consists of a constitutively expressed GR, which depends on Hsp90 for activation, coupled with a plasmid carrying a glucocorticoid response element driving *lacZ* (GRE-*lacZ*). Treatment of yeast cells containing the plasmids with deoxycorticosterone (DOC, a synthetic activator of GR) resulted in an approximate 10-fold induction in β-Gal activity in cells treated with DMSO only (Fig. 4D). In contrast, treatment with increasing concentrations of PUUP blocked the hormone-dependent induction of the reporter (Fig. 4D). We also observed this inhibition of reporter activity in the presence of celastrol (Fig. S4B). To verify that the observed effects are Hsp90 dependent, we made use of a plasmid that carries a truncated version of GR that excludes the ligand-binding domain and functions as an Hsp90-independent constitutive activator (34). In cells containing this plasmid along with the GRE-*lacZ* plasmid, high levels of β-Gal activity were observed in the presence of DMSO under –DOC as well as +DOC conditions (Fig. S4B). This induction was not inhibited in the presence of PUUP or celastrol, indicating that the two compounds specifically interfere with Hsp90 activity (Fig. S4B).

Finally, we made use of yeast overexpression strains to verify the effect of PUUP on Hsp90-related functions. Because the overexpression of heat shock proteins, their cochaperones, and client proteins have been implicated in the development of resistance to Hsp90 inhibitors (35), we were interested in determining if the overexpression of these proteins would result in resistance to PUUP. Therefore, we overexpressed genes encoding Hsp82 and Hsc82 (the two yeast Hsp90 isoforms), Cdc37 (an Hsp90 cochaperone), and Slt2 (an Hsp90 client protein) in a wild-type yeast strain (36–38). When we tested the sensitivity of the resulting strains to PUUP, they showed reduced sensitivity to PUUP compared to the wild-type strain (Fig. 4E), further confirming that PUUP inhibits Hsp90-related functions.

### Molecular docking of PUUP with Hsp90.

**FIG 5:**
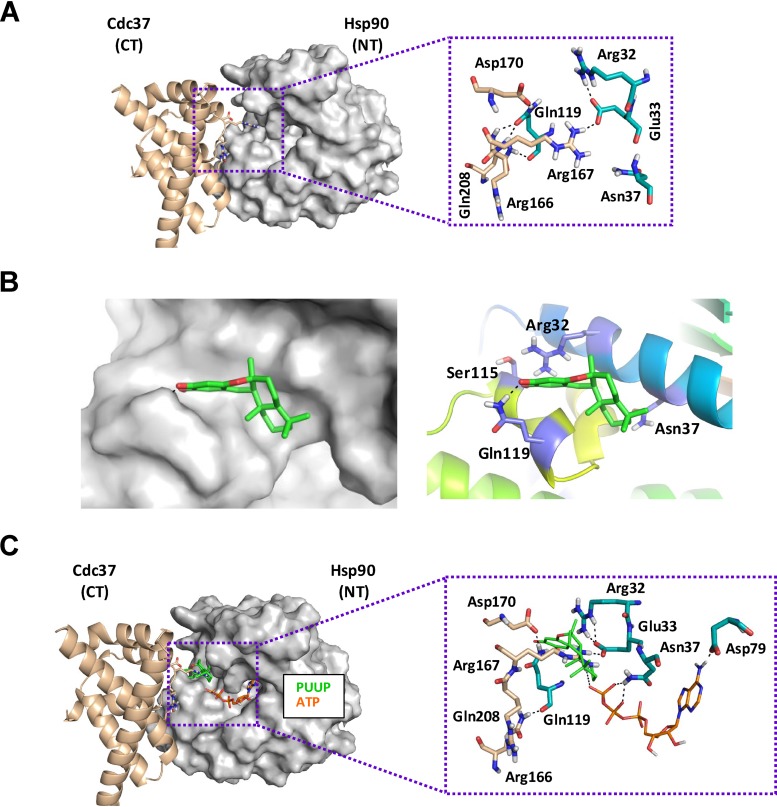
Molecular docking of PUUP with Hsp90 in the Hsp90-Cdc37 complex. (A) Hsp90-Cdc37 X-ray structure (PDB ID 1US7) depicting interactions between key residues in the N terminus (NT) of Hsp90 and the C terminus (CT) of Cdc37. The Cdc37 residues are shown as sticks with beige-colored carbons, while Hsp90 residues are shown as sticks with cyan-colored carbons. (B) Detailed depiction of the binding mode of PUUP to Hsp90 at the Hsp90-Cdc37 interaction site. The image on the left displays the shape of the binding pocket around the docked ligand. The image on the right shows the three-dimensional (3D) orientations of the residues interacting with the best docked pose of the ligand. (B) Overlay of the binding mode of PUUP (green carbon sticks) to Hsp90, along with the located ATP (orange carbon sticks) in the nucleotide binding pocket (conformation extracted from PDB ID 2CG9).

To explore potential binding interactions between PUUP and Hsp90, *in silico* molecular docking studies were conducted (Fig. 5). Given the mechanistic and structural similarities shared between PUUP and the Hsp90 inhibitor celastrol (Fig. S1), we first used the known interactions between celastrol and Hsp90 (39) to guide these efforts. One of the mechanisms by which celastrol is known to disrupt Hsp90 function is by blocking interaction with its cochaperone Cdc37 (39, 40). Significantly, the Hsp90-Cdc37 complex is required for the activation of client protein kinases such as Slt2 (37), and given that Slt2 activation was inhibited by PUUP (Fig. 3B) and that strains either lacking *SLT2* (Fig. 4B) or overexpressing *SLT2* and *CDC37* (Fig. 4E) displayed altered sensitivity to PUUP, it is reasonable to speculate that PUUP could similarly interfere with Hsp90-Cdc37 interactions.

To assess whether PUUP could potentially bind Hsp90 residues located at the interface of the Hsp90-Cdc37 complex, molecular docking was performed based on the available X-ray crystal structure of this complex (PDB identifier [ID] 1US7) (41). Structural analysis of the Hsp90-Cdc37 complex has previously revealed that the Gln-119 residue of Hsp90 (Fig. 5A) exhibits strong polar interactions with the Gln-208, Arg-166, and Arg-167 residues of Cdc37 (41). Our molecular docking results predicted that PUUP binds within the polar pocket of Hsp90 surrounded by Gln-119, Arg-32, and Asn-37 and directly interacts with Gln-119 and Ser-115 (Fig. 5B). We performed a similar docking experiment between celastrol and Hsp90-Cdc37, and the model obtained predicted binding within the same polar pocket of Hsp90 (Fig. S5), consistent with results reported by Zhang et al. (39). Our model also predicted similar binding affinities for PUUP and celastrol with Hsp90 (XP-Glide Score of −4.397 for PUUP and −4.933 for celastrol). Given that celastrol disrupts Hsp90-Cdc37 complex formation (40), our model strongly supports the hypothesis that PUUP could inhibit Hsp90-Cdc37 complex formation in a similar manner.

10.1128/mSphere.00818-19.5 FIG S5 Molecular docking of celastrol (CELA) with Hsp90 in the Hsp90-Cdc37 complex. (A) Detailed depiction of the binding mode of CELA to Hsp90 at the Hsp90-Cdc37 interaction site. The image on the left displays the shape of the binding pocket around the docked ligand. The image on the right shows the 3D orientations of the residues interacting with the best docked pose of the ligand. (B) Overlay of the binding mode of CELA (green carbon sticks) to Hsp90, along with the located ATP (orange carbon sticks) in the nucleotide binding pocket (conformation extracted from PDB ID 2CG9). Download FIG S5, PDF file, 0.3 MB. Copyright © 2020 Tripathi et al. 2020 Tripathi et al. This content is distributed under the terms of the Creative Commons Attribution 4.0 International license.

In contrast to celastrol, the Hsp90 inhibitors geldanamycin and radicicol have been shown to interact with the protein’s N-terminal ATP-binding site (42); therefore, we were also interested in examining potential interactions between this Hsp90 domain and PUUP. To accomplish this, we made use of the crystal structure (PDB ID 2CG9) available for Hsp90 complexed with ATP and the cochaperone Sba1 (43). We extracted the conformation of the Hsp90-ATP complex from this structure and then docked PUUP with the resulting complex. The resulting model, however, did not support a prediction of binding by PUUP to the N-terminal ATP-binding site, located in proximity to Gln-119 and the polar pocket of Hsp90 (Fig. 5C). A similar docking experiment was performed with celastrol, and the results from this analysis also failed to predict interaction within the Hsp90 nucleotide binding site (Fig. S5), consistent with previous observations that celastrol does not competitively inhibit nucleotide binding to Hsp90 (39). Taken together, the molecular docking experiments point to the Hsp90 polar pocket near Gln-119 as the likely target site for PUUP, which could potentially impair CWI pathway signaling by inhibiting Hsp90-Cdc37 interactions.

## DISCUSSION

**FIG 6:**
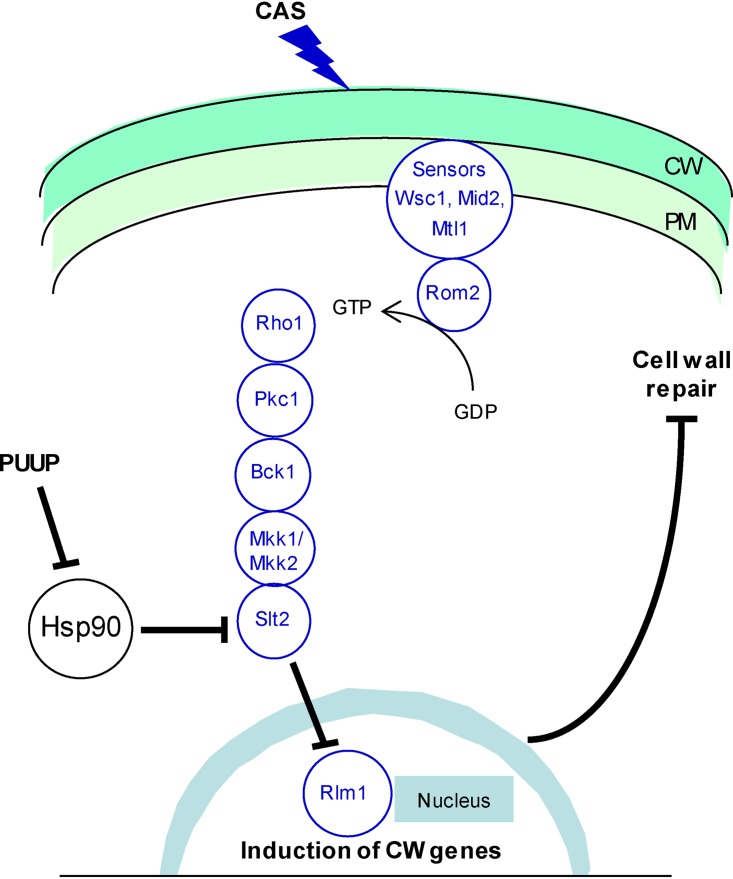
Model illustrating the putative mechanism by which PUUP potentiates CAS activity. The CWI pathway is initiated when cell wall (CW) damage caused by CAS is sensed by cell surface sensors in the plasma membrane (PM). The CW stress signal is relayed to the nucleus through a MAP kinase cascade, and the induction of CW-related genes leads to cell wall repair. PUUP interferes with Hsp90 function, resulting in the disruption of Slt2 activation. This in turn prevents the activation of Rlm1 and inhibits the induction of genes required for CW repair and maintenance.

In the work presented here, we show that the marine-sponge-derived sesquiterpene quinone PUUP potentiates the activity of CAS, a clinically used antifungal drug, by a mechanism that involves the disruption of Hsp90 activity and the CWI signaling pathway. We suggest that in this CAS-potentiating mechanism, the inhibition of Hsp90 activity disrupts the activation of the Hsp90 client protein Slt2, and this disruption inhibits the induction of Rlm1, ultimately blocking the process of cell wall repair due to the misregulation of cell wall biogenesis genes (Fig. 6).

Several pharmacological inhibitors have been reported to synergize with the echinocandin class of antifungals, with the most-well-characterized ones being chitin synthase inhibitors, calcineurin inhibitors, and Hsp90 inhibitors (6, 44). The mechanisms responsible for the synergistic effects of these inhibitors are different from the mechanism we have identified for PUUP. The combination of echinocandins and chitin synthase inhibitors causes severe damage to the fungal cell wall by depleting both glucan and chitin from the cell wall (44). Calcineurin inhibitors synergize with echinocandins due to the fact that calcineurin plays an important role in mediating cellular stress responses in fungal cells. Calcineurin is a protein phosphatase which regulates the transcription factor Crz1, which in turn is required for the activation of genes involved in cell wall maintenance and ion homeostasis in several fungal species (45). In C. albicans, for example, calcineurin also plays a role in adaptation to the cell wall stress exerted by echinocandins (44, 46). Treatment of C. albicans with echinocandins results in a compensatory increase in chitin synthesis, which is regulated, in part, by the calcineurin signaling pathway (47). Therefore, calcineurin inhibitors show a strong synergistic interaction with echinocandins in C. albicans (48). Similarly, the synergistic effects between echinocandins and Hsp90 inhibitors are attributable to the fact that Hsp90 is an important molecular chaperone in fungal cells and stabilizes several client proteins that are required for adaptation to cellular stresses (49). Mechanistic studies in C. albicans have revealed that Hsp90 depletion resulted in the destabilization of its client protein, calcineurin, and as mentioned above, calcineurin is required for cell wall stress adaptation. The Hsp90 inhibitor radicicol prevented the induction of a calcineurin-dependent stress response promoter in C. albicans that was activated by the echinocandin micafungin (48).

Interestingly, in contrast to our work with PUUP, the previous study by Singh et al. (48) did not report deactivation of the client protein kinase Mkc1 (the Slt2 homolog in C. albicans) in response to the Hsp90 inhibitor radicicol. It is worth noting that the micafungin-mediated activation of calcineurin-dependent stress response in the previous study was observed after 8 h of treatment with micafungin (48), whereas the CAS-mediated phosphorylation of Slt2 has been shown to occur quite rapidly, and we detected it within 15 min of CAS treatment (Fig. 3B). In support of this observation, we found that the CAS-mediated induction of a calcineurin-dependent stress response promoter was not detectable after 4 h of CAS treatment; however, it was detected after 12 h of CAS treatment, and this induction was blocked by PUUP (Fig. S6). Thus, it is possible that the deactivation of Slt2 (or Mkc1) due to Hsp90 inhibition is an early effect and the deactivation of calcineurin is a delayed effect. It is also possible that a compound such as PUUP which could potentially disrupt Hsp90-Cdc37 interaction would strongly interfere with the activation of Slt2, given that Cdc37 is the main cochaperone involved in recruiting protein kinase client proteins to Hsp90 (17). Collectively, our results suggest that inhibiting the interaction between Hsp90 and Cdc37 would be a useful approach for disrupting the CWI pathway and potentiating the activity of echinocandin drugs in recalcitrant organisms. One potential advantage of this approach would be that the association between Hsp90 and Cdc37 could be inhibited by compounds that target either protein. Since cochaperones such as Cdc37 are more divergent between fungi and humans than are Hsp90 isoforms (49), specific inhibitors of fungal Cdc37 could provide a means to circumvent the toxic effects of Hsp90 inhibitors in the potentiation of echinocandin antifungals.

10.1128/mSphere.00818-19.6 FIG S6 Effect of CAS and CAS+PUUP on the calcineurin-dependent stress response. β-Gal activity was measured in yeast cells containing a plasmid carrying the *lacZ* reporter driven by the calcineurin-dependent response element (CDRE) (Stathopoulos and Cyert [62]) after the cells were treated with DMSO, CAS, or CAS+PUUP for 4 h or 12 h. DMSO treatment was at 0.5%, and compound treatments were at their respective IC_50_s (0.016 μg/ml for CAS and 0.7 μg/ml for PUUP). Values shown are the mean ± SD from triplicate samples. CAS-mediated induction of CDRE-*lacZ* was observed after cells were exposed to CAS for 12 h, and this induction was inhibited by CAS+PUUP. (A) β-Gal activity measured after a 4-h drug exposure. (B) β-Gal activity measured after a 12-h drug exposure. Download FIG S6, PDF file, 0.08 MB. Copyright © 2020 Tripathi et al. 2020 Tripathi et al. This content is distributed under the terms of the Creative Commons Attribution 4.0 International license.

The present results demonstrate that PUUP exerts its biological effects by interfering with Hsp90 activity. This conclusion is based on the following lines of evidence: (i) the transcript and fitness profiles of PUUP were similar to those of Hsp90 inhibitors, (ii) PUUP activated Hsf1, (iii) PUUP blocked GR activation, (iv) yeast strains overexpressing genes encoding Hsp90 isoforms, an Hsp90 client protein, and an Hsp90 cochaperone were less sensitive to PUUP, and (v) molecular modeling predicted that PUUP binds to Hsp90 at a site involved in Hsp90-Cdc37 interactions. Given the limited number of compounds available which block the interaction between Hsp90 and Cdc37 (50), and with the availability of synthetic schemes for the synthesis of PUUP and its derivatives (51), PUUP will undoubtedly serve as an important pharmacological tool in the further characterization of Hsp90-Cdc37 interactions in pathogenic fungi and other eukaryotic organisms.

Clinical resistance to echinocandins is associated with mutations in the *FKS* genes encoding the catalytic subunits of glucan synthase, the enzymatic target of echinocandins (4, 52). Adaptive cellular responses, which are induced due to the cell wall stress exerted by echinocandins, play an important role in the development of these mutations. These adaptive responses cause the stabilization of a population of cells, allowing them time to transiently withstand drug action and eventually generate *FKS* mutations (4, 52). The CWI signaling pathway is the major cell wall stress adaptation pathway in fungal cells, and genes encoding most of its components have been identified in several fungal pathogens, including C. albicans, C. glabrata, and C. neoformans (53). In C. albicans, strains carrying deletions in *PKC1*, *BCK1*, *MKK2*, and *MKC1* are highly susceptible to CAS (54). In addition, cercosporamide, an inhibitor of Pkc1, potentiates the activity of an echinocandin analog in C. albicans (55). The CWI pathway also plays a role in the compensatory increase in chitin synthesis in C. albicans (47), and elevated chitin levels have been detected in clinical isolates of C. albicans with mutations in *FKS1* (56). Thus, pharmacological inhibitors of the CWI pathway would be useful in modulating the susceptibility to echinocandins in C. albicans. In C. glabrata, mutants with deletions in *BCK1*, *MKK1*, *SLT2*, *RLM1*, and *FKS1* show increased sensitivity to CAS (57). A few echinocandin-resistant C. glabrata strains with *FKS* mutations have been found to exhibit increased expression of the *FKS1* gene (58), which is regulated by the CWI pathway. Thus, CWI pathway inhibitors may be useful in preventing as well as overcoming resistance to echinocandins in *C glabrata*. In C. neoformans, CAS treatment causes the phosphorylation of Mpk1 (the Slt2 homolog in C. neoformans), and even though this organism is inherently insensitive to echinocandins, mutant cells lacking Mpk1 show increased sensitivity to CAS (59). Thus, inhibitors of the CWI pathway can cause echinocandins to become effective against this pathogen. In summary, pharmacological inhibition of the CWI pathway can be used to improve the efficacy of echinocandins in C. albicans, C. glabrata, and C. neoformans. In the work presented here, we have identified PUUP as a compound that disrupts this pathway and shown that it enhances the effectiveness of CAS against drug-resistant strains of C. albicans and C. glabrata and against C. neoformans. Our mechanistic studies show that PUUP inhibits the CWI pathway by interfering with Hsp90 function, potentially by disrupting Hsp90-Cdc37 interaction. While additional studies will be required to determine the precise mechanism by which PUUP disrupts Hsp90-Cdc37 interaction, this work lays the groundwork for the future utilization of compounds such as PUUP in echinocandin-based combination therapies for the treatment of difficult-to-treat fungal infections.

## MATERIALS AND METHODS

### Strains, media, and chemicals.

The strains used in this study are listed in Table S3. Synthetic dextrose (SD) medium, containing 0.67% yeast nitrogen base (without amino acids) and 2% dextrose, was used to grow the wild-type S. cerevisiae S288C strain. The medium was buffered with 0.165 M morpholinepropanesulfonic acid (MOPS), and the pH was adjusted to 7.0. For plasmid-containing strains, dropout media, with no pH adjustment, were used to maintain plasmid selection. RPMI 1640 medium buffered with MOPS to pH 7 was used for the dose matrix assays on fungal pathogens, except for assays with C. albicans, in which case the medium was buffered with MOPS to pH 6. For the genome-wide fitness profiling test, the haploid selection synthetic medium was prepared as described previously (26). CAS was obtained from Sigma-Aldrich. Puupehenone and sapindoside A were obtained from the compound repository of the National Center for Natural Products Research at the University of Mississippi. All stock solutions were made in DMSO.

10.1128/mSphere.00818-19.9 TABLE S3 Strains and plasmids used in this study. Download Table S3, PDF file, 0.1 MB. Copyright © 2020 Tripathi et al. 2020 Tripathi et al. This content is distributed under the terms of the Creative Commons Attribution 4.0 International license.

### Dose matrix assays.

Single colonies of the test organisms were grown overnight in RPMI 1640 medium. The cultures were diluted in broth after comparison to the 0.5 McFarland standard to afford a final inoculum of 1 × 10^4^ CFU/ml. The inoculum was added in a 180-μl volume to a microplate containing 10 μl of PUUP and 10 μl of CAS at various concentrations. CAS dilutions were placed in consecutive rows of the microplate and PUUP dilutions in consecutive columns. Appropriate controls included solvent control, medium control, a row of only CAS dilutions, and a column of only PUUP dilutions. The microplate was read at 600 nm prior to and after incubation at 35°C (for *Candida* strains) or 30°C (for the *Cryptococcus* strain) for 48 h, using a Tecan SPECTRAFluor Plus plate reader. To monitor cell viability, the microplate was shaken on an Eppendorf MixMate mixer, and a 2-μl aliquot from each well was spotted on a YPD agar plate, which was then incubated at 35°C (for *Candida* strains) or 30°C (for the *Cryptococcus* strain) for 24 h. The fractional inhibitory concentration index (FICI) was calculated with the equation FICI = [A*]/[A] + [B*]/[B], where [A*] is the IC_50_ of compound A in the presence of compound B, [A] is the IC_50_ of compound A alone, [B*] is the IC_50_ of compound B in the presence of compound A, and [B] is the IC_50_ of compound B alone. An FICI of ≤0.5 was considered to indicate a synergistic interaction.

### RNA-seq analysis.

An overnight culture of S. cerevisiae strain S288C, started from a single colony, was used to inoculate 50 ml of SD medium to an *A*_600_ of 0.1. Three replicate cultures were started for each treatment. After one doubling, cultures were treated with CAS (0.03 μg/ml), PUUP (0.87 μg/ml), or CAS+PUUP (0.03 μg/ml CAS plus 0.87 μg/ml PUUP), with the concentrations used corresponding to the IC_50_ value of each compound. Control cultures were treated with 0.5% DMSO at the same time. After drug treatment, the cultures were allowed to grow until an *A*_600_ of 0.5 was reached (∼4 h). Cells were harvested by centrifugation, flash frozen in liquid nitrogen, and stored at –80°C. RNA was isolated as described previously (12), and the isolated RNA was subjected to Qiagen’s on-column RNase-free DNase I treatment. The quality of the RNA samples was assessed on the Agilent 2100 Bioanalyzer, and all samples displayed an RNA integrity number (RIN) of >9.

Sequencing libraries were generated with 4 μg of RNA using the Illumina TruSeq stranded mRNA sample preparation kit. The libraries were assessed for size and purity using the Agilent 2100 Bioanalyzer and quantitated by quantitative PCR (qPCR) using a Kapa Biosystems library quantitation kit. Libraries were normalized, pooled, and diluted to a loading concentration of 1.8 pM, loaded on an Illumina mid-output flow cell, and sequenced on the Illumina NextSeq 500 instrument. The sequencing run generated ∼20 million 150-bp paired-end reads per sample, and all run metrics, including cluster densities and total sequence yields, were within the recommended parameters.

Data analysis was performed using the Qiagen CLC Genomics Workbench (CLCGxWB) version 11 software. Quality assessment of the reads indicated that the average median score at each base across the reads in each sample was Q > 30. The reads were mapped to the S. cerevisiae reference genome (R64-1-1 release), using default parameters with a strand-specific alignment protocol. The mapping report indicated that all metrics fell within the recommended parameters, with >80% of the reads per sample mapping to the reference genome. The mapping results generated values for fragments per kilobase of exon per million reads mapped (FPKM) for each gene. The FPKM values were used to generate a PCA plot with the CLCGxWB software to assess the relatedness among the samples. The software was also used to perform hierarchical cluster analysis with normalized FPKM values, using Euclidean distance and complete linkage settings. The “Differential Expression for RNA-seq” tool in the CLCGxWB software was used, using default parameters, to identify differentially expressed genes between drug-treated and solvent-treated samples. Genes that had a fold change of ≥2, a Bonferroni-corrected *P* value of <0.01, and a maximum group mean FPKM of >5 (i.e., the maximum of the average FPKM across the two groups in each statistical comparison) were considered to be significantly differentially expressed. The list of differentially expressed genes responding to each treatment is shown in Table S2. The GO Term Mapper tool (https://go.princeton.edu/cgi-bin/GOTermMapper) was used to distribute PUUP-responding genes into GO-based biological process categories.

### Genome-wide fitness profiling analysis.

This analysis was carried out essentially as previously described (26). Briefly, a pool of haploid-convertible heterozygote diploid yeast deletion mutants was sporulated. Pools of isogenic *MAT***a** haploid cells were derived by growth for 2 days on a haploid selection medium (SC-Leu-His-Arg plus G418 and canavanine) that either contained or lacked PUUP. The compound concentration used was 2 μg/ml, resulting in partial growth inhibition of the *ho*Δ mutant, which served as a surrogate wild-type control. The relative representation of each deletion mutant in drug-treated and untreated pools was compared by tag array analysis. For validation, individual haploid convertible heterozygous diploid mutants were sporulated, spotted onto haploid selection medium with PUUP at a subinhibitory concentration, and incubated at 30°C for 3 days.

The similarity between the fitness profile of PUUP and those reported for elevated temperature, geldanamycin, and macbecin II was assessed using Fisher’s exact test, in which we compared the proportion of mutants overlapping the PUUP data set with the proportion of mutants reported to be hypersensitive in each of three studies (27–29). The *P* values obtained were <0.0001 for the elevated temperature and geldanamycin comparisons and 0.01992 for the macbecin II comparison. Applying the Holm method (60), the *P* values of the three individual tests were ordered from smallest to largest, *P1, P2*, and* P3*, where both *P1* and *P2* were <0.0001 and *P3* was 0.01992. With the significance level α set at 0.02, all three comparisons were found to be statistically significant since they followed the expected trend of *P1* < α/3, *P2* < α/2, and *P3* < α. Thus, the overlap with the PUUP data set was statistically significant in the three comparisons at a significance level (α) of 0.02.

### Western blot analysis.

An overnight culture of S. cerevisiae strain S288C was grown in SD broth (MOPS buffered [pH 7.0]) and used to inoculate fresh medium at an *A*_600_ of 0.1. Four cultures were started for each treatment, in duplicate. After one doubling, CAS, PUUP, CAS+PUUP, or DMSO (0.5%) was added to the cultures at the respective IC_50_ of each compound. The cells were harvested by centrifugation at 15 min, 30 min, 60 min, and 120 min after treatment. Two independent experiments were performed on independently grown cultures. Protein extracts were prepared by treating the cell pellets with yeast protein extract reagent (Y-PER; Thermo Fisher), according to the manufacturer’s instructions, with the modification that 1% β-mercaptoethanol (BME), 10 mM phenylmethylsulfonyl fluoride (PMSF), and 2× Thermo Scientific Halt phosphatase inhibitor cocktail were added to the Y-PER. Protein concentration was determined using the Bradford assay. Fifty micrograms of protein from each extract was separated by SDS-PAGE, followed by Western blotting. Phosphorylated Slt2 was detected by using an anti-phospho-p42/44 MAP kinase (Thr^202^/Tyr^204^) antibody (Cell Signaling, Danvers, MA) at a 1:2,000 dilution. A monoclonal antibody to yeast phosphoglycerate kinase (Pgk1) (Thermo Fisher) was used as a loading control. Immunoblots were developed using the Amersham ECL Select kit, and images were captured on the Bio-Rad ChemiDoc MP imaging system.

### Yeast overexpression strain construction and susceptibility testing.

Four genes involved in Hsp90-related functions (*HSP82*, *HSC82*, *CDC37*, and *SLT2*) were cloned into the 2 μ plasmid pRS426 using the Clontech In-Fusion HD cloning kit (TaKaRa Bio USA, Mountain View, CA). Gene-specific primers were designed to include the coding region as well as approximately 1,000 bp upstream and 300 bp downstream of the coding region (Table S4). To each primer, 15-bp extensions were added that overlapped the plasmid termini generated when digested with the BamHI and HindIII enzymes. PCRs were performed with yeast genomic DNA as the template using the reagents provided in the cloning kit. The PCR fragment generated was fused to the plasmid (digested with BamHI and HindIII) by homologous recombination, using the reagents from the cloning kit. The plasmid was transformed into competent Escherichia coli cells provided in the cloning kit. Sequence-confirmed clones were transformed into competent yeast cells (strain BY4742) using the lithium acetate method (61). To determine the sensitivities of the yeast strains to PUUP, agar-based drop assays were performed. Overnight cultures of the four strains and the parent strain BY4742 were grown in SC broth (SD medium containing 0.77% complete synthetic mixture). The cultures were diluted to an *A*_600_ of 3.0, and serial dilutions (1:5) were prepared in SC broth. The dilutions were spotted in 3-μl amounts on SC agar plates containing 1% DMSO or 6 μg/ml PUUP, a concentration that caused partial growth inhibition of the parent strain on SC agar plates. The plates were incubated for 3 days at 30°C.

10.1128/mSphere.00818-19.10 TABLE S4 List of primers used in this study. Download Table S4, PDF file, 0.1 MB. Copyright © 2020 Tripathi et al. 2020 Tripathi et al. This content is distributed under the terms of the Creative Commons Attribution 4.0 International license.

### Reporter assays.

All constructs utilized *lacZ* as the reporter, and the yeast β-Gal assay kit (Thermo Fisher) was used in all assays. All absorbance reads were measured on the Tecan SPECTRAFluor Plus microplate reader.

**(i) *MLP1-lacZ* assays.** An overnight culture from a single colony of a S. cerevisiae strain, harboring a plasmid expressing *lacZ* under the control of the *MLP1* (*KDX1*) promoter region (19), was grown in SC-URA (SC minus uracil) broth at 30°C. Two independent experiments were conducted with two independent colonies. The overnight culture was inoculated into fresh broth to achieve an *A*_600_ of 0.4. After a 2-h recovery, the cells were treated with DMSO (0.5%), CAS, PUUP, or CAS+PUUP at the IC_50_ value of each compound. The cells were transferred to a microplate, in 100-μl aliquots, in triplicate. After 2 h, 4 h, and 6 h of drug treatment, the *A*_600_ was measured on a plate reader to determine cell quantity, and 100 μl of a working solution (WS) was added to the cells, which consisted of equal volumes of Y-PER and the β-Gal assay buffer provided in the assay kit. A kinetic run was performed at *A*_420_, with reads measured every 2 min. The β-Gal activity of each sample was calculated in Miller units, as per the instructions in the assay kit.

**(ii) *HSE-lacZ* assays.** An overnight culture from a single colony of a S. cerevisiae strain harboring an HSE-*lacZ* plasmid (32) was grown in SC-URA broth at 30°C. Two independent experiments were conducted with two independent colonies. The overnight culture was inoculated into fresh broth to achieve an *A*_600_ of 0.4. Four cultures with a 5-ml volume each were started for four different treatments. After a 2-h recovery, cultures were treated with either PUUP (0.83, 1.66, and 3.32 μg/ml) or DMSO (0.25%). After 4 h of drug treatment, aliquots of cells were removed from each culture, pelleted by centrifugation, and adjusted to an *A*_600_ of 4.0 with broth. To each cell aliquot, an equal volume of WS buffer was added, and the mixture was incubated at 37°C until a color change was observed. The reaction was stopped using the β-Gal assay stop buffer provided in the assay kit. After a brief centrifugation, the supernatant was transferred to a microplate in triplicate. The *A*_420_ was measured, and the β-Gal activity was calculated.

**(iii) Glucocorticoid receptor system assays.** An overnight culture from a single colony of an S. cerevisiae strain, harboring plasmids p413GPD-rGR and pYRP-GRE-*lacZ* (33), was grown in SC-URA-HIS (SC minus uracil and histidine) broth at 30°C. Two independent experiments were conducted with two independent colonies. The overnight culture was inoculated into fresh broth to achieve an *A*_600_ of 6.0. Eight cultures of 1 ml each were started for four different treatments in duplicate. The duplicate cultures were treated with either PUUP (0.83, 1.66, and 3.32 μg/ml) or DMSO (0.25%). For each treatment, 20 μM deoxycorticosterone (DOC; Sigma) was added to one of the duplicate cultures, and the vehicle ethanol (0.5% final concentration) was added to the other culture. After 2 h of drug treatment, aliquots of cells were removed from each culture and adjusted to an *A*_600_ of 6.0 with broth. To each cell aliquot, an equal volume of WS buffer was added and incubated at 37°C until a color change was observed. The reaction was stopped using the stop buffer provided in the assay kit. After a brief centrifugation, the supernatant was transferred to a microplate in triplicate. The *A*_420_ was measured, and β-Gal activity was calculated.

### Growth assay with yeast cells expressing *MKK1-S386P*.

Yeast strains, harboring either a plasmid with wild-type *MKK1* or a plasmid with the mutant allele *MKK1-S386P* fused to the *GAL1* promoter (20), were grown overnight in SC-URA broth (contains glucose as the carbon source) at 30°C. The cells were inoculated into SG-URA broth (contains galactose as the carbon source) to achieve an *A*_600_ of 0.05 to 0.1. Cells were treated with various concentrations of PUUP (16 ng/ml to 250 ng/ml) or sapindoside A (SAPI; 0.5 μg/ml to 8 μg/ml) and allowed to grow for 6 h. The *A*_600_ values were measured, from triplicate samples, and plotted against compound concentrations.

### Molecular docking studies.

The X-ray crystal structure of the Hsp90-Cdc37 complex (41) was downloaded from the Protein Data Bank website (PDB ID 1US7). Since the ATPase activity of Hsp90 is inhibited by Cdc37 through interaction between the Arg-167 residue of Cdc37 and the Glu-33 residue of Hsp90 (41), and since this interaction is inhibited by celastrol (39), the Glu-33 residue of Hsp90 was defined as the centroid residue for the docking studies. PUUP and celastrol were docked to Hsp90, using the Induced Fit Docking protocol implemented in the Small Molecule Drug Discovery Suite release 2013-1 available in the Schrödinger software platform (Schrödinger, LLC, New York, NY). Per-residue analysis was performed using the Extra Precision (XP) method of Glide Docking in the Schrödinger platform to further specify the binding interactions between the docked ligands and residues within 5 Å of the ligand. Binding poses were scored using XP-GlideScores. Graphics were created using the PyMOL program available in the Schrödinger platform. To determine if PUUP binds to the ATP-binding site of Hsp90, molecular modeling was performed based on the X-ray crystal structure of Hsp90 N-terminal domain (NTD) in complex with ATP and the cochaperone Sba1 (PDB ID 2CG9) reported by Ali et al. (43). The Hsp90(NTD)-ATP complex was extracted from this structure, and PUUP and celastrol were docked to the resulting complex.

### Data availability.

The RNA-seq analysis data described in this article are accessible through accession number GSE140563 at the NCBI’s Gene Expression Omnibus database.
