# Supplementary figures and images for: Puupehenone, a Marine-Sponge-Derived Sesquiterpene Quinone, Potentiates the Antifungal Drug Caspofungin by Disrupting Hsp90 Activity and the Cell Wall Integrity Pathway
Source: mSphere. 2020 Jan 8;5(1):e00818-19. doi: 10.1128/mSphere.00818-19 (PMC6952202; doi:10.1128/mSphere.00818-19)

**A**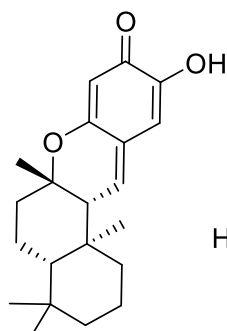

Puupehenone

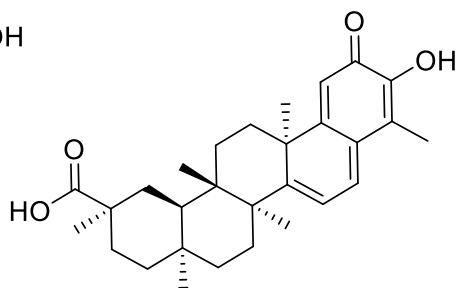

Celastrol

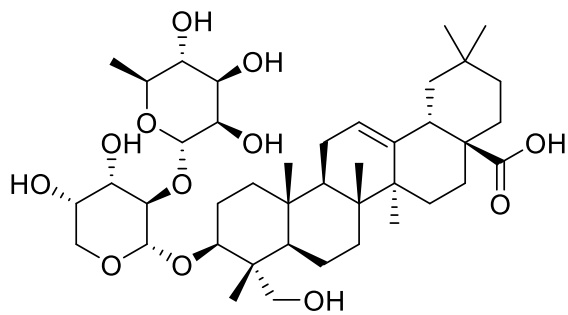

Sapindoside A

**B**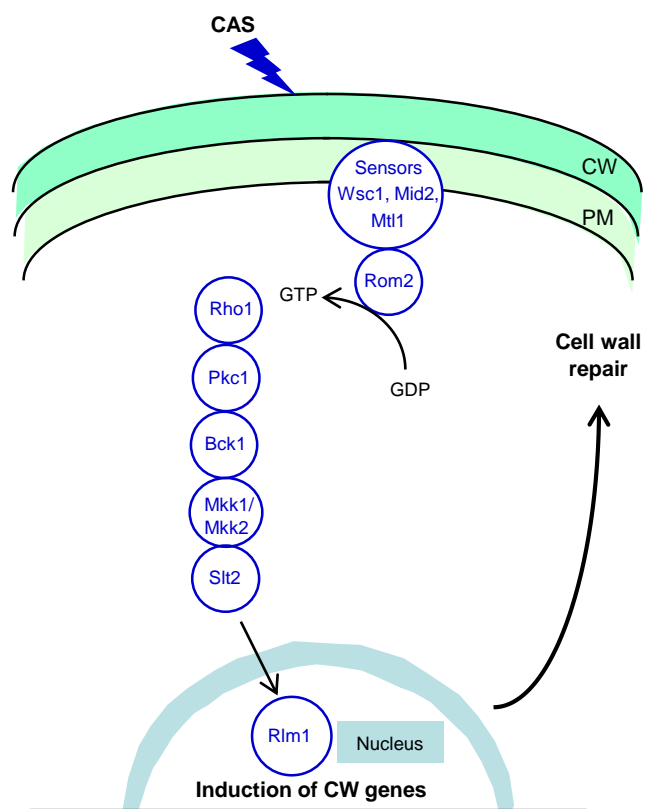

Figure S1

Supplement: FIG S1 [file mSphere.00818-19-sf001.pdf]

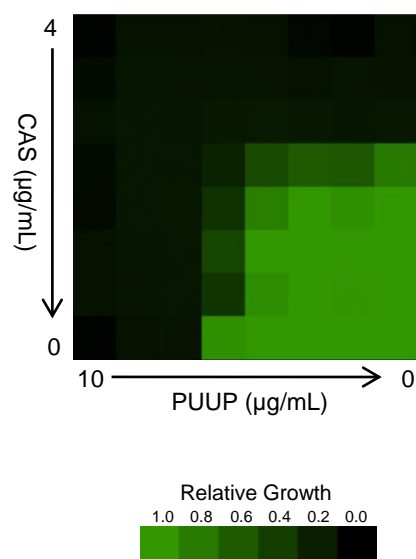

**Figure S2**

Supplement: FIG S2 [file mSphere.00818-19-sf002.pdf]

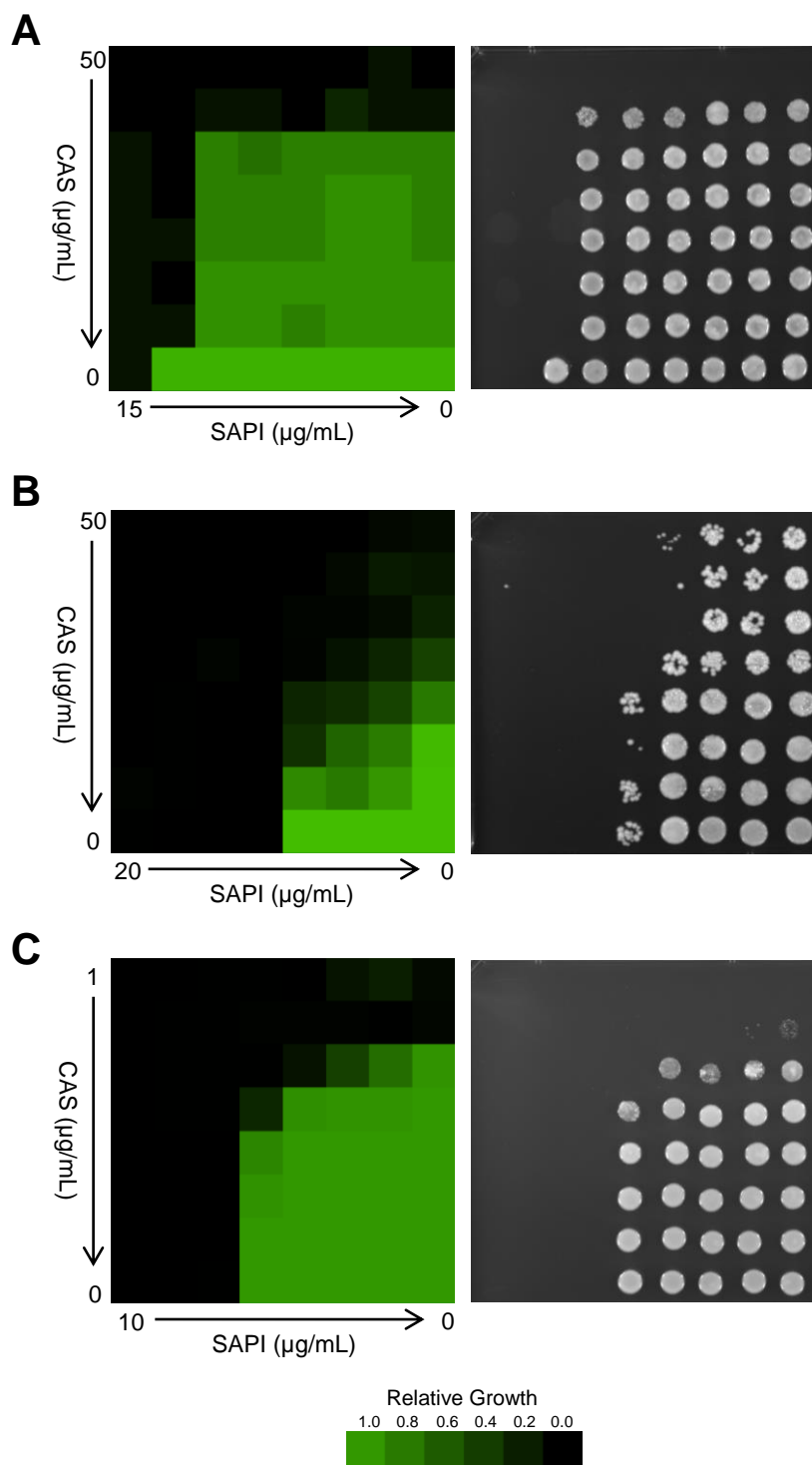

**Figure S3**

Supplement: FIG S3 [file mSphere.00818-19-sf003.pdf]

**A**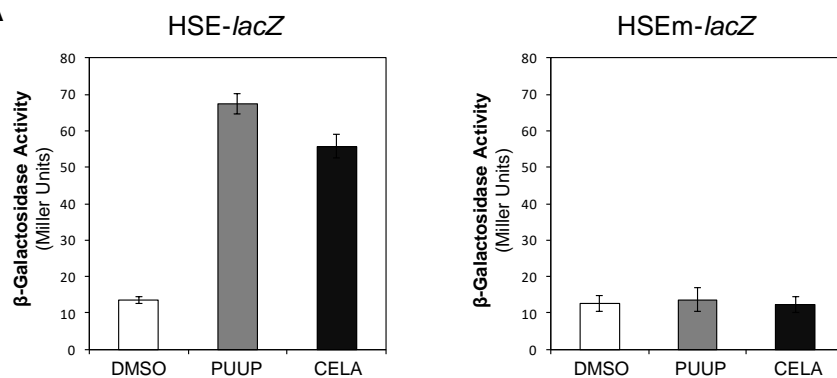**B**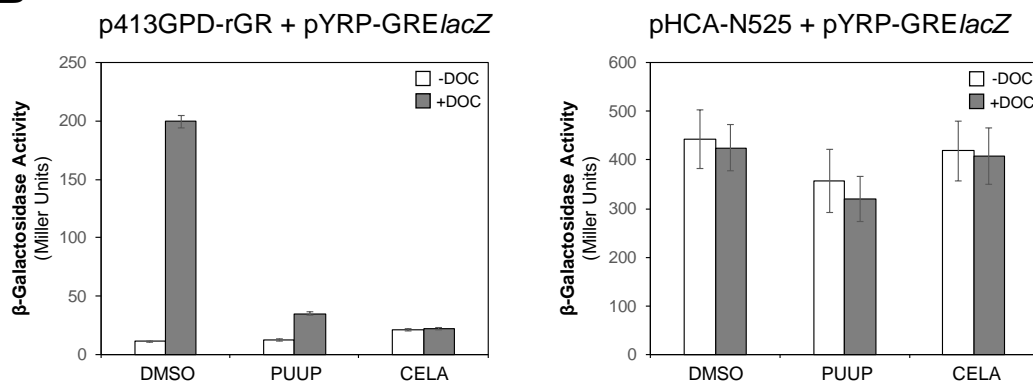**Figure S4**

Supplement: FIG S4 [file mSphere.00818-19-sf004.pdf]

**A**

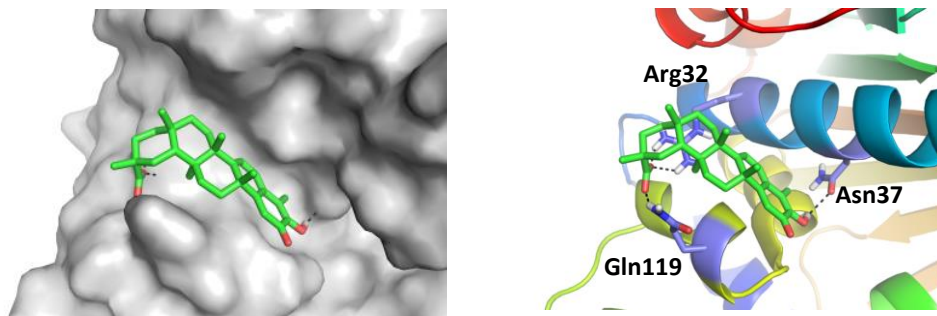

**B**

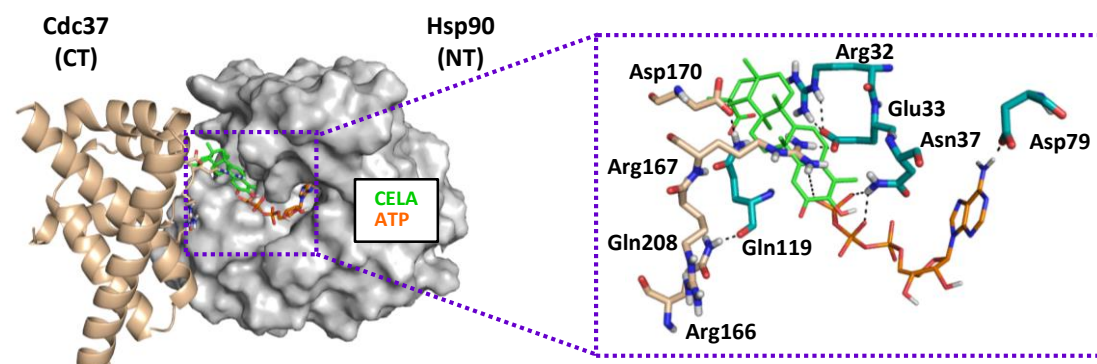

**Figure S5**

Supplement: FIG S5 [file mSphere.00818-19-sf005.pdf]

**A**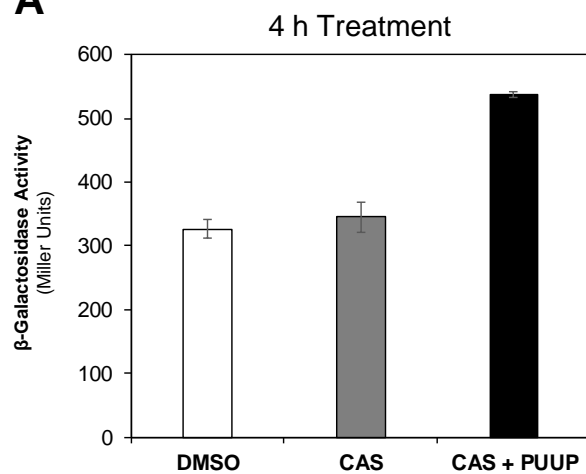**B**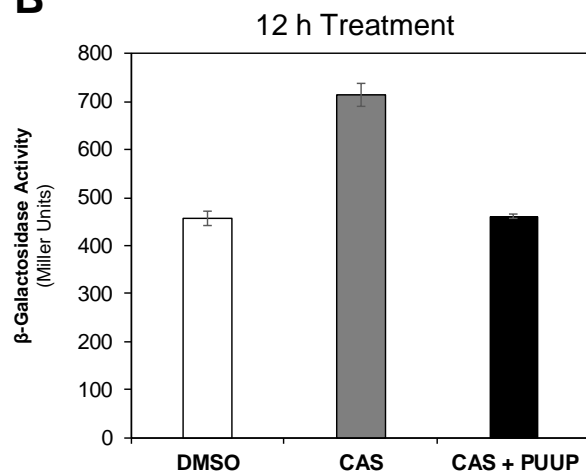

**Figure S6**

Supplement: FIG S6 [file mSphere.00818-19-sf006.pdf]
