# Supplementary material for: Puupehenone, a Marine-Sponge-Derived Sesquiterpene Quinone, Potentiates the Antifungal Drug Caspofungin by Disrupting Hsp90 Activity and the Cell Wall Integrity Pathway
Source: mSphere. 2020 Jan 8;5(1):e00818-19. doi: 10.1128/mSphere.00818-19 (PMC6952202; doi:10.1128/mSphere.00818-19)
Supplement: TABLE S1 [file mSphere.00818-19-st001.pdf]

Supplemental Table S1. Fractional Inhibitory Concentration Index Values for Dose-Matrix Assays

Organism	IC_{50} of CAS[a]	IC_{50} of CAS with PUUP	IC_{50} of PUUP	IC_{50} of PUUP with CAS	FICI[b]
Cryptococcus neoformans (strain H99)	30[c]	1.5	0.6	0.2	0.38
Candida glabrata (strain 102)	17	2	0.72	0.26	0.48
Candida albicans (strain DPL1010)	20	6	3.8	0.35	0.39

[a] IC_{50} values are based on dose response curves.

[b] FICI = [A*]/[A] + [B*]/[B], where[A*] is IC_{50} of compound A in the presence of compound B, [A] is IC_{50} of compound A alone, [B*] is IC_{50} of compound B in the presence of compound A, and [B] is IC_{50} of compound B alone.

[c] All values are in μg/ml.
