# Supplementary material for: Puupehenone, a Marine-Sponge-Derived Sesquiterpene Quinone, Potentiates the Antifungal Drug Caspofungin by Disrupting Hsp90 Activity and the Cell Wall Integrity Pathway
Source: mSphere. 2020 Jan 8;5(1):e00818-19. doi: 10.1128/mSphere.00818-19 (PMC6952202; doi:10.1128/mSphere.00818-19)
Supplement: TABLE S3 [file mSphere.00818-19-st003.pdf]

Supplemental Table S3. Strains and Plasmids Used in this Study

Strain/Plasmid	Description	Source
Strains		
S288C	*MATα, SUC2, mal, mel, gal2, CUP1, flo1, flo8-1*	ATCC
BY4742	*MATα, his3Δ1, leu2Δ0, lys2Δ0, ura3Δ0*	Open Biosystems, AL
STSc042	BY4742 containing plasmid p1368	This study
STSc035	BY4742 containing plasmid HSE-*lacZ*	This study
STSc036	BY4742 containing plasmid HSEm-*lacZ*	This study
STSc033	BY4742 containing plasmids p413GPD-rGR and pYRP-GRE-*lacZ*	This study
STSc038	BY4742 containing plasmids pHCA-N525 and pYRP-GRE-*lacZ*	This study
QFSc058	BY4742 containing plasmid QFEc058	This study
QFSc059	BY4742 containing plasmid QFEc059	This study
QFSc060	BY4742 containing plasmid QFEc060	This study
QFSc061	BY4742 containing plasmid QFEc061	This study
STSc063	W303-1A containing plasmid pAM366	Xu et al., 2011, AAC, 55: 1611
DL102	*MAT*a/α, *his4/-, leu2-3,112/-, trp1-1/-, ura3-52/-, can1R/-*	David Levin, Boston Univ.
DL102, pMKK1-WT	DL102 containing a plasmid expressing the WT version of *MKK1*	David Levin, Boston Univ.
DL102, pMKK1-S386P	DL102 containing a plasmid expressing a mutant version of *MKK1* with S386P mutation	David Levin, Boston Univ.
CnH99	*Cryptococcus neoformans* (strain H99)	ATCC
Cg102	*Candida glabrata* (clinical isolate number 102 with *FKS2* F659del mutation)	Ryan Shields, U. of Pittsburgh
CaDPL1009	*Candida albicans* (clinical isolate number 1009 with *FKS1* S645Y mutation)	David Perlin, Rutgers Univ.
CaDPL1010	*Candida albicans* (clinical isolate number 1010 with *FKS1* S645F mutation)	David Perlin, Rutgers Univ.
Plasmids		
p1368	*MLP1* promoter fused to *lacZ*	David Levin, Boston Univ.
HSE-*lacZ*	*SSA3* promoter fused to *lacZ*	Kevin Morano, UT Houston
HSEm-*lacZ*	*SSA3* promoter mutated at -156 fused to *lacZ*	Kevin Morano, UT Houston
p413GPD-rGR	Glyceraldehyde-3-phosphate dehydrogenase promoter fused to glucocorticoid receptor (GR)	Kevin Morano, UT Houston
pYRP-GRE-*lacZ*	Glucocorticoid receptor element fused to *lacZ*	Kevin Morano, UT Houston
pHCA-N525	Plasmid expressing truncated glucocorticoid receptor (up to N525)	Kevin Morano, UT Houston
pAMS366	4X-CDRE fused to *lacZ*	Stathopoulos et al., 1997, Genes Dev., 11: 3432
QFEc058	pRS426 plasmid containing *S. cerevisiae CDC37* (1000 bp 5' + ORF + 300 bp 3')	This study
QFEc059	pRS426 plasmid containing *S. cerevisiae HSC82* (1000 bp 5' + ORF + 300 bp 3')	This study
QFEc060	pRS426 plasmid containing *S. cerevisiae HSP82* (1000 bp 5' + ORF + 300 bp 3')	This study
QFEc061	pRS426 plasmid containing *S. cerevisiae SLT2* (1000 bp 5' + ORF + 300 bp 3')	This study
