# Supplementary material for: Puupehenone, a Marine-Sponge-Derived Sesquiterpene Quinone, Potentiates the Antifungal Drug Caspofungin by Disrupting Hsp90 Activity and the Cell Wall Integrity Pathway
Source: mSphere. 2020 Jan 8;5(1):e00818-19. doi: 10.1128/mSphere.00818-19 (PMC6952202; doi:10.1128/mSphere.00818-19)
Supplement: TABLE S4 [file mSphere.00818-19-st004.pdf]

Supplemental Table S4. List of Primers Used in this Study

Gene	Primer Name	Primer Sequence	Amplicon Size (bp)
CDC37	CDC37-INF-F1	5'-CGGTATCGATAAGCTTTCGCAGAAAACATGAAGAAGAAGC-3'	2961
	CDC37-INF-R1	5'-TAGAACTAGTGGATCCAGCGTAGCAAGTTCTACTAATGGCG-3'	
HSC82	HSC82-INF-F1	5'-CGGTATCGATAAGCTTACTTACAACGCTATGTGAATTAGAGC-3'	3267
	HSC82-INF-R1	5'-TAGAACTAGTGGATCCCTAAACTTTAAGGACAGCTGGTAGGA-3'	
HSP82	HSP82-INF-F1	5'-CGGTATCGATAAGCTTCTTTCAGAGAATCCAAATCGCCAG-3'	3435
	HSP82-INF-R1	5'-TAGAACTAGTGGATCCGAGTTCTCGTTCGAGCTCTACAC -3'	
SLT2	SLT2-INF-F1	5'-CGGTATCGATAAGCTTGTCATGGACATGAGATTAGTGAGTCG-3'	2756
	SLT2-INF-F1	5'-TAGAACTAGTGGATCCAGGTACCATTAAAGTCATATTCTAAGCG-3'	

Sequences in red indicate 15-bp extensions which are homologous to the cut plasmid ends
Underlined sequence represents restriction sites; HindIII: AAGCTT; BamHI: GGATCC
